# Sensitive Period Analysis of Adulthood BMI and Cancer Risk: An Individual Participant Data Meta‐Analysis of Over 720,000 Participants in the ABACus 2 Consortium

**DOI:** 10.1002/ijc.70464

**Published:** 2026-04-08

**Authors:** Nadin K. Hawwash, Matthew Sperrin, Glen P. Martin, Rashmi Sinha, Charles E. Matthews, Matthias B. Schulze, Anouk Hiensch, Pilar Amiano, Marian L. Neuhouser, Corinne E. Joshu, Elizabeth A. Platz, Heinz Freisling, Marc J. Gunter, Andrew G. Renehan

**Affiliations:** ^1^ Division of Cancer Sciences, School of Medical Sciences, Faculty of Biology, Medicine and Health University of Manchester Manchester UK; ^2^ Cancer Research UK Manchester Cancer Research Centre Manchester UK; ^3^ Centre for Health Informatics, Division of Informatics, Imaging and Data Sciences, School of Health Sciences, Faculty of Biology, Medicine and Health University of Manchester Manchester UK; ^4^ Metabolic Epidemiology Branch, Division of Cancer Epidemiology and Genetics National Cancer Institute Shady Grove USA; ^5^ Department of Molecular Epidemiology German Institute of Human Nutrition Potsdam‐Rehbruecke Nuthetal Germany; ^6^ Institute of Nutritional Science University of Potsdam Nuthetal Germany; ^7^ Julius Center for Health Sciences and Primary Care, University Medical Center Utrecht Utrecht University Utrecht the Netherlands; ^8^ Spanish Consortium for Research on Epidemiology and Public Health (CIBERESP) Instituto de Salud Carlos III Madrid Spain; ^9^ Ministry of Health of the Basque Government Sub Directorate for Public Health and Addictions of Gipuzkoa San Sebastian Spain; ^10^ Fred Hutchinson Cancer Center Seattle Washington USA; ^11^ Department of Epidemiology Johns Hopkins Bloomberg School of Public Health Baltimore Maryland USA; ^12^ Nutrition and Metabolism Branch International Agency for Research on Cancer (IARC‐WHO) Lyon France; ^13^ Cancer Epidemiology and Prevention Research Unit, School of Public Health Imperial College London London UK; ^14^ National Institute for Health Research (NIHR) Manchester Biomedical Research Centre Manchester UK

**Keywords:** cancer, life course, obesity, prediction, sensitive periods analysis

## Abstract

At least 13 cancers are linked to obesity. We analyse time‐to‐event data using Sensitive Period Analysis to explore whether associations between body mass index (BMI) and cancer incidence vary throughout adulthood to inform cancer prevention strategies, policy and weight management trials of optimal intervention ages. Using the European Prospective Investigation into Cancer and Nutrition cohort, Atherosclerosis Risk in Communities study, Women's Health Initiative, Prostate, Lung, Colorectal, Ovarian Cancer Screening Trial, NIH‐AARP Diet and Health, we predicted BMI throughout adulthood. We landmarked to predefined ages of interest (AOI), ages 30 to 65 (5‐yearly). Super‐landmarking and a two‐stage IPD meta‐analysis were used. A single stratified Cox proportional hazards model with interaction terms between BMI and AOIs was fitted to analyse associations between per 5 kg/m^2^ BMI at AOIs and cancer incidence and identify sensitive age periods. 720,210 participants were followed up over 9.85 years in men and 10.80 years in women. Positive associations were found per 5 kg/m^2^ BMI across ages 30–65 for obesity‐related cancers. Some evidence suggests BMI in the 40s–50s raises cancer risk more than baseline. Interactions by age were found in women at ages 35 and 40 for obesity‐related cancers with HRs per 5 kg/m^2^ of 1.04 (95% CI: 1.01, 1.07, *I* ^2^:0%) and 1.05 (95% CI: 1.01, 1.09, *I* ^2^:50%), respectively, and at ages 35–65 for postmenopausal breast cancer. Higher BMI increased obesity‐related cancer risk across ages 30–65. Similar associations across adulthood suggest adiposity at any age increases cancer risk. Policymakers should prevent excess adiposity accumulation in early life to minimise cancer risk.

## Abstract (highlights)

What's new?

Over a dozen cancers have been associated with excess body mass index, primarily in mid‐to‐late adulthood. Whether obesity‐related cancer risk differs across adulthood remains unclear. Using the Sensitive Periods Model and super‐landmarking approaches, this study found that higher BMI increased obesity‐related cancer risk across ages 30–65. BMI–cancer associations in the fourth and fifth decades of life seemed to contribute more to cancer risk than associations at other ages. Similar associations across adulthood however suggested that adiposity at any age increases cancer risk. The findings strengthen the rationale to implement targeted prevention strategies early in adulthood to minimise BMI exposure.

## Graphical abstract

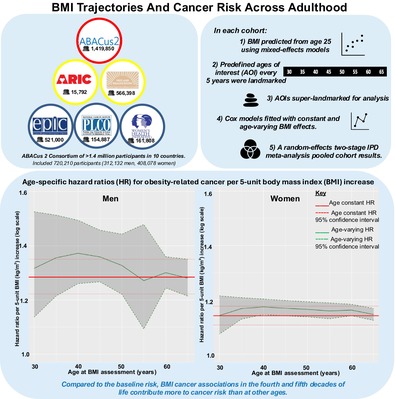

**Abbreviations**
AOI: age of interest
ARIC: Atherosclerosis Risk in Communities study
BMI: body mass index
CI: confidence interval
EPIC: European Prospective Investigation into Cancer and Nutrition study
HR: hazard ratio
IPD: individual participant data
LA: landmark analysis
MV: multivariable
NIH‐AARP: NIH‐AARP Diet and Health Study
NOBR: non‐obesity‐related cancers
OBR: obesity‐related cancers
PLCO: Prostate, Lung, Colorectal, Ovarian Cancer Screening Trial
SPA: sensitive period analysis
WHI: Women's Health Initiative

## 1 Introduction

The obesity epidemic is a major global health concern with rates dramatically rising in low‐ and middle‐income countries [1]. By 2035, around 51% globally will be overweight or obese [2]. Over 13 cancers are associated with excess body mass index (BMI) primarily found on analysis of participants in mid‐to‐late adulthood [3]. One question is whether obesity‐cancer risk differs across adulthood. Briefly, 15 studies explored the BMI‐cancer link at particular ages (Table S1) with 12 studies analysing the interaction of age [4, 5, 6, 7, 8, 9, 10, 11]. Two analysed BMI‐cancer associations at specific ages using an individual participant data (IPD) meta‐analysis, but did not analyse for interactions by age [12, 13]. One study analysed BMI‐related premenopausal breast cancer risk across adulthood using a fixed effect IPD meta‐analysis; however, there were variations across cohorts, such as differential tumour type classification [10]. Another study found no BMI‐cancer associations for advanced prostate cancer risk in early adulthood but associations in mid‐to‐late adulthood, demonstrating the importance of analysis by age [13]. Studies may be subject to immortal time bias—guaranteed outcome‐free survival periods between age of BMI exposure and follow‐up starting [14]. Although studies explored adiposity timing across the life course and cancer risk, insufficient evidence remains of magnitude and patterns of variation in obesity‐cancer associations by age that consider cohort‐ and individual‐level variations. Understanding this will identify sensitive obesity‐cancer age periods which may aid hypotheses of etiological relevance and design age‐targeted cancer prevention programmes which minimise cancer incidences.

We applied sensitive period analysis (SPA) to analyse BMI‐cancer associations at ages throughout adulthood whilst accounting for immortal time bias [3]. A random effects two‐stage IPD meta‐analysis across large cohorts will account for cohort‐ and individual‐level variations. Cohorts with larger proportions of racial groups will be analysed to overcome prior limitations (Table S1).

The Sensitive Periods Model focuses on exposure timing and risk of disease [15]. Landmark analysis (LA) is traditionally used to condition outcomes to future assigned landmark time, allowing for updated outcome prediction [14]. We propose LA use in SPA. In SPA, the exposure status is captured at predefined biologically and clinically meaningful ages or multiple ages of interest (AOIs) and associations with the outcome are modelled throughout follow‐up with landmarking [14]. Figure S1a hypothetically demonstrates no interactions of age on BMI and cancer. Figure S1b shows a temporal association where the age‐varying hazard ratio (HR) varies from the age‐fixed HR. SPA identified whether BMI at AOIs (ages 30–65, 5‐yearly) has stronger or weaker associations than other ages with cancer incidence at any time in the future to generate hypotheses on mechanistic insights and contribute to clinical decision‐making. Further details regarding the evidence before this study, its added value and the implications of all available evidence can be found in the Supporting Information.

## 2 Methods

### 2.1 Study Population

We assembled the ABACus 2 Consortium of > 1.4 million participants from Women's Health Initiative (WHI) [16] and Prostate, Lung, Colorectal, Ovarian Cancer Screening Trial (PLCO) [17], NIH‐AARP Diet and Health Study (NIH‐AARP) [18], Atherosclerosis Risk in Communities study (ARIC) [19], and European Prospective Investigation into Cancer and Nutrition (EPIC) study [20] (Table S2). Men and women were analysed separately [21].

### 2.2 Eligibility Criteria

Participants > 80 years old at study entry, with at least 3 BMI measurements, obtained retrospectively or prospectively, with cancer at or before the AOI and/or with BMI ≤ 15 kg/m^2^ and ≥ 60 kg/m^2^ were excluded.

### 2.3 Exposure

The exposure of interest was predicted BMI weight (in kg) divided by height squared (in metres) at ages 30–65 in increments of 5 years in 5 kg/m^2^ (Table S2) to avoid data‐driven selection bias and capture general age‐related trends. Ages 30–65 were analysed, given the availability of data across all cohorts. BMI at age 25 was used as a reference group in this study.

### 2.4 Outcome

Primary cancer incidence was the outcome of interest. Obesity‐related cancers included colorectal, gastric, oesophageal, thyroid, kidney, liver, pancreatic, multiple myeloma, gallbladder, meningioma in men and additionally breast, endometrial and ovarian cancers in women [4]. Non‐obesity‐related cancers were cancers beyond these. In EPIC, non‐melanoma skin cancers were excluded, and non‐melanoma skin cancer is likely to be uncollected or underreported in the other cohorts. Follow‐up ended with the first primary cancer diagnosis, end of cancer follow‐up or death, whichever occurred first. Cancer‐specific sites with ≥ 10 events per candidate predictor parameter were analysed separately. Therefore, we analysed colorectal, pancreas, kidney, lung, endometrial (in women), ovary (in women), postmenopausal breast (in women) and prostate cancers (in men) separately.

### 2.5 Covariates

Covariates at study entry adjusted for were smoking as ‘ever smokers’ and ‘never smokers’, race as ‘White’, ‘Black’, and ‘Other’ (non‐White and non‐Black participants), hormone replacement therapy (HRT) as ‘ever HRT users’ and ‘never HRT users’ and alcohol (units/week).

### 2.6 Statistical Analysis

**FIGURE 1:**
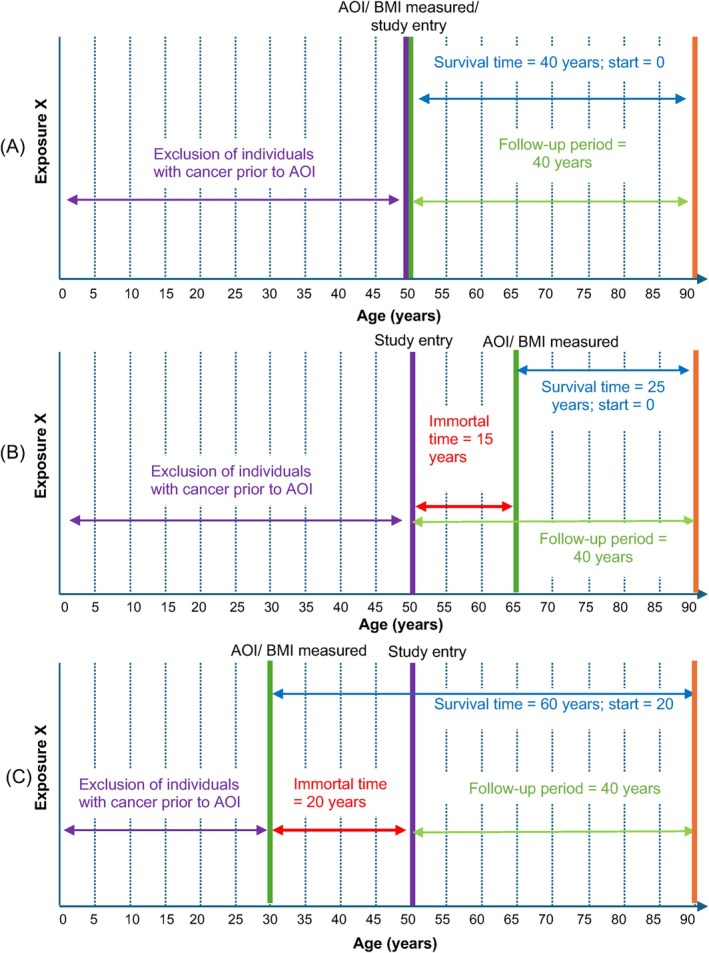
Demonstrating sensitive period analysis (SPA) when the age of interest (AOI) is (A) at the index date (B) after the index age, and (C) SPA when the AOI is before the index date. BMI measurements at the AOI were the exposure of interest. The AOIs were ages 30–65 in increments of 5. The survival time is the period during which participants are considered at risk of cancer. The follow‐up period is the period during which participants were observed for cancer incidence. The immortal period is a period where a participant is guaranteed survival and is cancer‐free. In (A) survival time is equal to the follow‐up period. In (B) survival time is equal to follow‐up time minus the immortal time, and the start time is 0. In (C) the survival time is equal to the immortal time added to the follow‐up period, and the start time is the difference in years between AOI and study entry.

We conducted a two‐stage random‐effects IPD meta‐analysis. Multiple imputation with predictive mean matching imputed missing covariate data, generating 10 imputed datasets per cohort [22]. ‘Race’, ‘alcohol’, ‘smoking’, ‘education’, ‘HRT’, ‘cancer incidence’, ‘age of cancer diagnosis’, ‘ever diagnosed with diabetes’, ‘ever diagnosed with heart disease’ variables were included in the predictor matrix. Missing BMI measurements were not imputed. First, yearly BMI across adulthood was retrospectively predicted from the index date of each cohort using the available observed, self‐reported or recalled BMI measurements, whichever was present in each cohort study, as shown in Table S2. BMI was predicted per year using a mixed‐effects model with a random intercept and slope on a participant level with a spline on age to allow for non‐linear trends in BMI over age. An interaction term on sex as a fixed effect on age predicted BMI. BMI was predicted for each participant per year from the minimum age BMI was recalled/collected/measured until cancer incidence/administrative censoring/death. Second, each cohort was landmarked at each AOI. Each cohort was subset to only include data for an AOI (ages 30 to 65 5‐yearly) and included participants that survived and were cancer‐free [23]. Eight datasets per cohort for each AOI were created. Third, survival time was defined (Figure 1). If AOI was at study entry and start of follow‐up, survival time equalled follow‐up (Figure 1A). If AOI was after study entry, survival time was immortal time subtracted from follow‐up (Figure 1B). If AOI was before study entry, survival time was immortal time added to follow‐up and left truncation accounted for the immortal period (Figure 1C). Fourth, super‐landmarking occurred by stacking datasets, each based on a particular AOI, clustering by participant ID and fitting (i) a single AOI stratified multivariable‐adjusted. As participants could contribute person‐times to multiple AOIs, we accounted for within‐person correlation using linear mixed‐effects models with participant‐level random effects. Specifically, models were fitted with the lme4::lmer()function, including random intercepts and random slopes for age at the participant level. Confidence intervals were derived directly from the mixed effects model. Cox proportional hazards regression model with a constant HR for continuous per 5 kg/m^2^ BMI and (ii) a multivariable‐adjusted Cox model with constant hazard for continuous per 5 kg/m^2^ BMI and an age‐varying hazard included with an interaction term between BMI and AOI. HRs of the interaction by age were obtained to identify the direction and significance of the interaction at each AOI. Fifth, HRs from each imputed dataset were pooled under Rubin's rules [24]. Sixth, cohort outcomes were meta‐analysed [25].

### 2.7 Sensitivity Analysis

A sensitivity analysis was completed comparing the HRs for cancer per 5‐unit BMI change at the AOIs throughout adulthood using the same multivariable adjustment as the main analysis but with one model adjusting for smoking stratified as ever or never, and another model quantifying smoking cumulatively through pack‐years, that is, the number of packs of cigarettes smoked per day multiplied by the number of years the person has smoked. Also, we further repeated analyses on participants with ≥ 1 observed BMI to identify selection bias towards healthier participants. PRISMA checklist was used (Table S3). R4.1.2 (RRID:SCR_001905). PROSPERO CRD42021238270.

## 3 Results

Briefly, 720,210 participants (43% men; 57% women) were included (Figure S2). The median age was 60.9 (interquartile range (IQR):59.5, 63.8) in men and 63.0 (IQR: 61.1, 66.9) in women, and the mean BMI was 27.2 and 26.9 kg/m^2^ respectively (Table S4). Observed and predicted BMI values are shown in Figure S3; 85,341 men and 63,732 women were diagnosed with cancer over a median follow‐up period of 9.85 years (IQR: 8.03, 11.67) and 10.80 years (IQR: 6.05, 15.55) respectively (Table S5); 64%–74% of men and 44%–55% of women were ever smokers, and 28%–67% of women were ever HRT users. Black participants ranged between 2% and 20% for men and 5% and 28% for women.

### 3.1 Associations Between BMI and Cancer Over Adulthood

**FIGURE 2:**
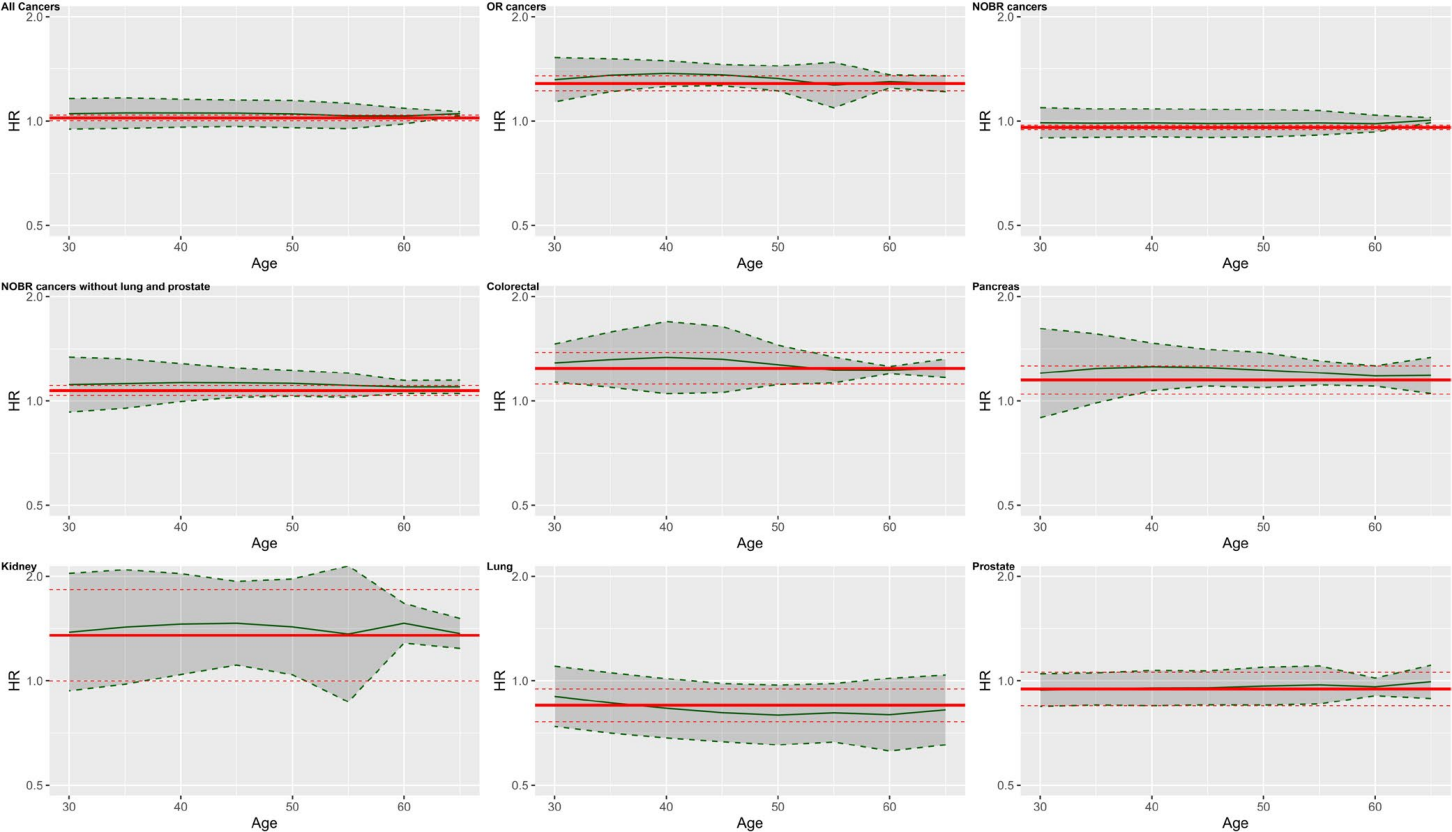
Hazard ratios of cancers per 5‐unit BMI for men for combined cancer subgroups and by cancer type over adulthood, in ABACus 2. The red line is the age‐constant hazard ratio (95% CI: Dashed red lines), and the green line is the age‐varying hazard, which includes an interaction between BMI and age (95% CI: Grey ribbon). The *x*‐axis refers to the age at BMI assessment. The *y*‐axis is on a log scale. Multivariable adjustment for baseline age, race, alcohol and smoking. OBR, obesity‐related cancers; NOBR, non‐obesity‐related cancers.

**FIGURE 3:**
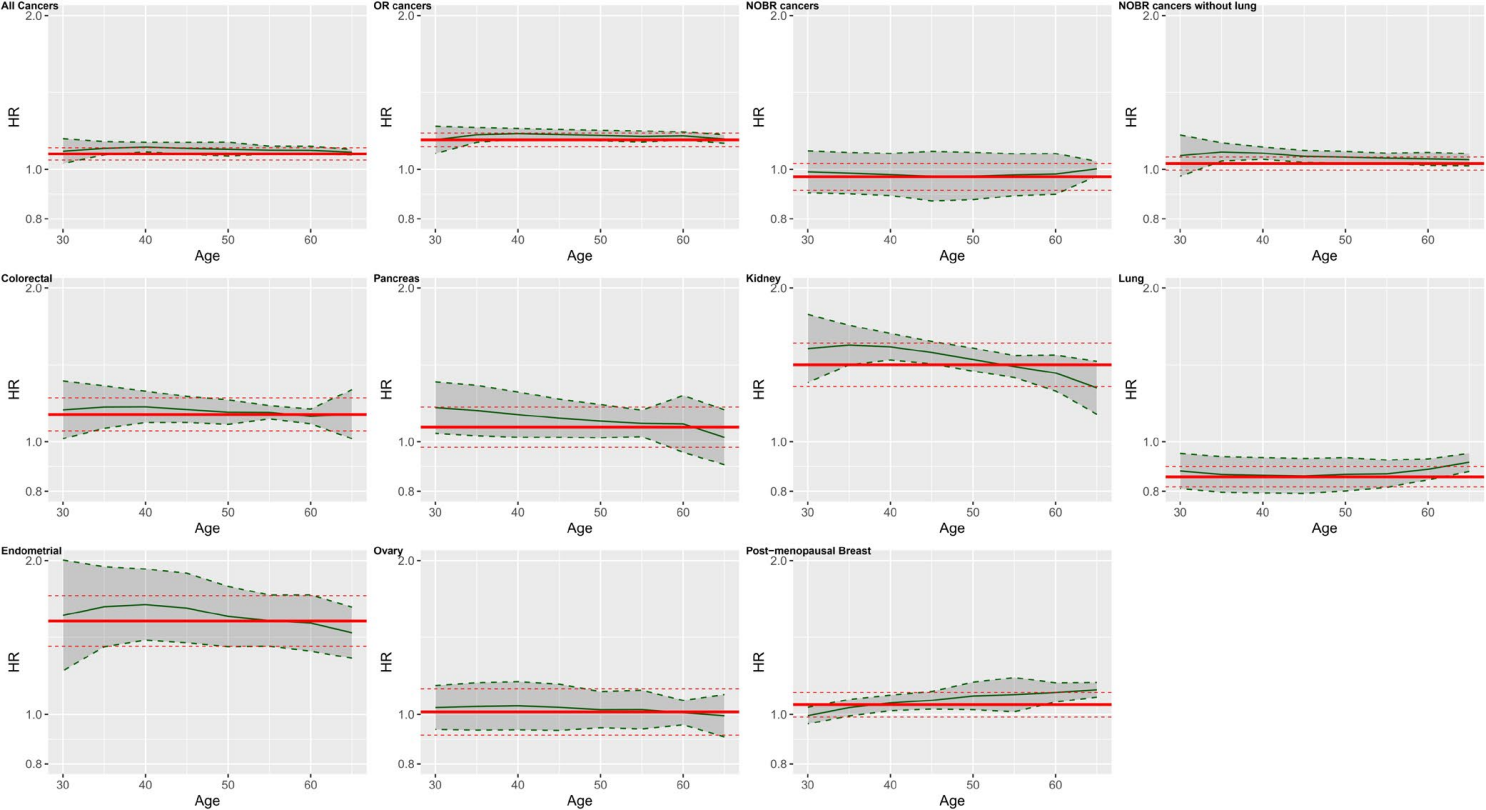
Hazard ratios of cancers per 5‐unit BMI for women for combined cancer subgroups and by cancer type over adulthood, in ABACus 2. The red line is the age‐constant hazard ratio (95% CI: Dashed red lines), and the green line is the age‐varying hazard, which includes an interaction between BMI and age (95% CI: Grey ribbon). The *x*‐axis refers to the age at BMI assessment. The *y*‐axis is on a log scale. Multivariable adjustment for baseline age, race, alcohol, smoking and hormone replacement therapy (in women). OBR, obesity‐related cancers; NOBR, non‐obesity‐related cancers; CI, confidence interval.

Across all cancers, positive associations per 5 kg/m^2^ increment in BMI across ages 30–65 for obesity‐related cancers in men and women were found. There is some evidence to suggest that, compared to the baseline risk, BMI cancer associations in the fourth and fifth decades of life contribute more to obesity‐related cancer risk than at other ages. Overall, there was considerable overlap between age‐constant and age‐varying HRs per 5 kg/m^2^ BMI at each AOI (Figures 2 and 3).

In men, for obesity‐related cancers, associations were positive across all AOI with HRs at ages 40 and 65 years of 1.37 (95% CI: 1.26, 1.49, *I* ^2^:60%) and 1.28 (95% CI: 1.21, 1.35, *I* ^2^:0%) respectively (Table 1a). There were positive associations between per 5‐unit BMI exposure and colorectal and pancreatic cancer incidence in men across ages 30–65 and for kidney cancer across ages 40–65. For non‐obesity‐related cancers, there were no associations at ages 35–60 but on excluding lung and prostate cancers, associations across ages 45–65 were positive. For lung and prostate cancers, all associations were inverse (Table 1a). In women, for obesity‐related cancers, associations were positive across all AOIs with HRs at age 30 of 1.14 (95% CI: 1.07, 1.21, *I* ^2^:74%) and age 65 of 1.15 (95% CI: 1.12, 1.17, *I* ^2^: 0%) (Table 1b). Positive associations across ages 30–65 per 5 kg/m^2^ BMI were found for colorectal, pancreas, kidney and endometrial cancers in women. For ovarian cancer, there was no evidence of an association between BMI and cancer incidence. For postmenopausal breast cancer, the association with per 5 kg/m^2^ BMI exposure was positive at ages 40–65. In women, for non‐obesity‐related cancers excluding lung cancers, positive associations were found across all AOI except at age 30 (Table 1b). Associations for cancer incidence per 5‐unit BMI exposure at ages 30 to 65 by cohort separately across cancer types are shown in Figure S4.

**TABLE 1:** Hazard ratios for cancer incidence per 5‐unit BMI exposure at ages 30 to 65 in (a) men and (b) women in the subgroup with at least 3 BMI measurements, ABACus 2.

(a)																
	MV‐adjusted HR (95% CI) per 5‐unit BMI (kg/m^2^)*															
	Men															
	Age 30	*I* ^2^	Age 35	*I* ^2^	Age 40	*I* ^2^	Age 45	*I* ^2^	Age 50	*I* ^2^	Age 55	*I* ^2^	Age 60	*I* ^2^	Age 65	*I* ^2^
All cancers	1.05 (0.95, 1.16)	0.95	1.05 (0.95, 1.17)	0.95	1.05 (0.96, 1.16)	0.95	1.05 (0.97, 1.15)	0.95	1.05 (0.96, 1.15)	0.96	1.04 (0.95, 1.13)	0.96	1.03 (0.98, 1.09)	0.85	1.05 (1.04, 1.06)	0.00
OBR‐cancers	1.32 (1.14, 1.53)	0.79	1.36 (1.21, 1.51)	0.68	1.37 (1.26, 1.49)	0.60	1.36 (1.27, 1.46)	0.53	1.33 (1.22, 1.44)	0.67	1.27 (1.09, 1.48)	0.80	1.3 (1.24, 1.36)	0.00	1.28 (1.21, 1.35)	0.00
NOBR‐cancers	0.99 (0.89, 1.09)	0.94	0.99 (0.90, 1.08)	0.94	0.99 (0.90, 1.08)	0.94	0.98 (0.90, 1.08)	0.95	0.99 (0.90, 1.08)	0.95	0.99 (0.91, 1.07)	0.94	0.98 (0.93, 1.04)	0.81	1.01 (0.99, 1.02)	0.00
NOBR cancers excluding lung and prostate	1.11 (0.93, 1.34)	0.87	1.12 (0.95, 1.32)	0.85	1.13 (1.00, 1.28)	0.79	1.13 (1.02, 1.24)	0.77	1.12 (1.03, 1.23)	0.79	1.11 (1.02, 1.20)	0.74	1.10 (1.05, 1.15)	0.01	1.10 (1.05, 1.15)	0.00
Specific cancer sites																
Colorectal	1.29 (1.13, 1.46)	0.40	1.32 (1.10, 1.58)	0.60	1.33 (1.05, 1.7)	0.71	1.32 (1.06, 1.64)	0.69	1.27 (1.12, 1.45)	0.57	1.23 (1.13, 1.34)	0.43	1.23 (1.20, 1.26)	0.00	1.24 (1.17, 1.32)	0.00
Pancreas	1.38 (0.94, 2.04)	0.78	1.43 (0.98, 2.09)	0.76	1.46 (1.04, 2.04)	0.72	1.47 (1.11, 1.93)	0.68	1.43 (1.04, 1.97)	0.71	1.37 (0.87, 2.14)	0.76	1.47 (1.28, 1.67)	0.28	1.37 (1.24, 1.51)	0.00
Kidney	1.20 (0.89, 1.62)	0.55	1.24 (0.99, 1.56)	0.41	1.25 (1.07, 1.47)	0.11	1.25 (1.11, 1.41)	0.00	1.23 (1.09, 1.38)	0.00	1.20 (1.11, 1.3)	0.00	1.18 (1.1, 1.26)	0.00	1.18 (1.05, 1.33)	0.00
Lung	0.90 (0.74, 1.10)	0.77	0.86 (0.71, 1.06)	0.80	0.83 (0.68, 1.01)	0.84	0.81 (0.67, 0.98)	0.85	0.80 (0.65, 0.97)	0.88	0.81 (0.67, 0.98)	0.88	0.8 (0.63, 1.02)	0.86	0.82 (0.65, 1.04)	0.67
Prostate	0.94 (0.84, 1.05)	0.82	0.95 (0.85, 1.05)	0.81	0.95 (0.85, 1.07)	0.84	0.95 (0.85, 1.07)	0.82	0.97 (0.85, 1.09)	0.87	0.97 (0.86, 1.10)	0.87	0.96 (0.91, 1.02)	0.42	0.99 (0.89, 1.11)	0.62

(b)																
	MV‐adjusted HR (95% CI) per 5 unit BMI (kg/m^2^)*															
	Women															
	Age 30	*I* ^2^	Age 35	*I* ^2^	Age 40	*I* ^2^	Age 45	*I* ^2^	Age 50	*I* ^2^	Age 55	*I* ^2^	Age 60	*I* ^2^	Age 65	*I* ^2^
All cancers	1.09 (1.03, 1.15)	0.81	1.10 (1.07, 1.13)	0.57	1.11 (1.08, 1.13)	0.42	1.10 (1.07, 1.13)	0.61	1.09 (1.06, 1.13)	0.77	1.09 (1.07, 1.11)	0.27	1.09 (1.07, 1.11)	0.19	1.08 (1.07, 1.09)	0.00
OBR‐cancers	1.14 (1.07, 1.21)	0.74	1.17 (1.13, 1.21)	0.41	1.17 (1.15, 1.2)	0.16	1.17 (1.14, 1.2)	0.23	1.16 (1.14, 1.19)	0.33	1.16 (1.13, 1.19)	0.38	1.16 (1.14, 1.18)	0.00	1.15 (1.12, 1.17)	0.00
NOBR‐cancers	0.99 (0.90, 1.09)	0.76	0.98 (0.90, 1.08)	0.72	0.98 (0.89, 1.07)	0.70	0.97 (0.87, 1.08)	0.75	0.97 (0.87, 1.08)	0.76	0.98 (0.89, 1.07)	0.72	0.98 (0.89, 1.07)	0.66	1.00 (0.97, 1.04)	0.00
NOBR cancers excluding lung and prostate	1.06 (0.97, 1.17)	0.64	1.08 (1.04, 1.13)	0.22	1.08 (1.05, 1.11)	0.00	1.06 (1.03, 1.09)	0.00	1.06 (1.03, 1.08)	0.00	1.05 (1.03, 1.08)	0.00	1.05 (1.02, 1.08)	0.00	1.04 (1.02, 1.07)	0.00
Specific cancer sites																
Colorectal	1.16 (1.01, 1.32)	0.64	1.17 (1.06, 1.29)	0.49	1.17 (1.09, 1.26)	0.33	1.16 (1.09, 1.23)	0.23	1.14 (1.08, 1.21)	0.26	1.14 (1.11, 1.18)	0.00	1.12 (1.08, 1.16)	0.00	1.13 (1.01, 1.27)	0.59
Pancreas	1.17 (1.04, 1.31)	0.04	1.15 (1.03, 1.29)	0.14	1.13 (1.02, 1.25)	0.09	1.11 (1.02, 1.21)	0.00	1.10 (1.02, 1.18)	0.00	1.09 (1.02, 1.15)	0.00	1.08 (0.95, 1.23)	0.43	1.02 (0.90, 1.16)	0.17
Kidney	1.52 (1.31, 1.78)	0.35	1.55 (1.42, 1.69)	0.00	1.53 (1.45, 1.63)	0.00	1.50 (1.42, 1.57)	0.00	1.45 (1.38, 1.53)	0.00	1.40 (1.34, 1.47)	0.00	1.36 (1.26, 1.48)	0.00	1.28 (1.13, 1.44)	0.00
Lung	0.88 (0.81, 0.95)	0.29	0.86 (0.80, 0.94)	0.34	0.86 (0.79, 0.93)	0.39	0.86 (0.79, 0.93)	0.43	0.86 (0.80, 0.93)	0.46	0.87 (0.81, 0.92)	0.31	0.88 (0.84, 0.93)	0.00	0.91 (0.88, 0.95)	0.00
Endometrial	1.56 (1.22, 2.01)	0.93	1.62 (1.36, 1.95)	0.91	1.64 (1.40, 1.93)	0.90	1.61 (1.38, 1.89)	0.90	1.55 (1.36, 1.78)	0.90	1.53 (1.36, 1.71)	0.85	1.51 (1.33, 1.71)	0.82	1.44 (1.29, 1.62)	0.64
Ovary	1.03 (0.93, 1.14)	0.00	1.04 (0.93, 1.15)	0.19	1.04 (0.93, 1.16)	0.30	1.03 (0.93, 1.15)	0.35	1.02 (0.94, 1.11)	0.16	1.02 (0.94, 1.12)	0.24	1.01 (0.95, 1.06)	0.00	0.99 (0.90, 1.09)	0.00
Post‐menopausal breast	0.99 (0.96, 1.03)	0.00	1.03 (0.99, 1.07)	0.00	1.05 (1.02, 1.09)	0.07	1.07 (1.02, 1.11)	0.27	1.09 (1.02, 1.16)	0.62	1.09 (1.01, 1.18)	0.68	1.10 (1.06, 1.15)	0.46	1.12 (1.08, 1.15)	0.00

*Note*: Green—significant positive interaction. Orange—significant inverse interaction. NB: immortal time bias has been accounted for across all age periods of interest.

Abbreviations: BMI, body mass index; CI, confidence interval; HR, hazard ratio; MV, multivariable; NOBR, non‐obesity related; OBR, obesity‐related.

* Multivariable adjustment for baseline age, race, alcohol, smoking, and hormone replacement therapy (in women).

### 3.2 Analysis of Interactions by Age on the BMI‐Cancer Link Over Adulthood

There were no interactions by age across obesity‐related cancers in men. An interaction by age was found for lung cancer at ages 35 to 60 in men with inverse HRs of association per 5 kg/m^2^ BMI (Table 2a). In women, for obesity‐related cancers, there were interactions by age at ages 35 and 40 with HRs of association per 5 kg/m^2^ of 1.04 (95% CI: 1.01, 1.07, *I* ^2^:0%) and 1.05 (95% CI: 1.01, 1.09, *I* ^2^:50%), respectively. For combined non‐obesity‐related cancers in women, there was an interaction at age 40 with a HR per 5 kg/m^2^ of 0.96 (95% CI: 0.93, 0.99, *I* ^2^:6%) but not on excluding lung cancers. For colorectal, pancreatic, kidney, lung, endometrial and ovarian cancers, there were no interactions by age except for postmenopausal breast cancer, where there was an interaction by age with increasing associations per 5 kg/m^2^ from ages 35–65 (Table 2a). The *I* ^2^ values of the interaction by age on the BMI‐cancer link, measuring the total variability due to between‐cohort study heterogeneity in the ABACus2 Consortium, are listed in Table S6 and show heterogeneity primarily increased for ages 45 and beyond. HRs of the interaction by age on BMI‐cancer link in men and women by cohort separately across cancer types are shown in Tables S7–S11.

**TABLE 2:** Hazard ratios of interaction by age on BMI‐cancer link in men and women, ABACUS 2.

Outcomes	MV‐adjusted HR (95% CI)*							
	Age 30	Age 35	Age 40	Age 45	Age 50	Age 55	Age 60	Age 65
Men								
All cancers	0.99 (0.97, 1.01)	0.99 (0.97, 1.02)	0.99 (0.96, 1.03)	0.99 (0.96, 1.03)	0.99 (0.95, 1.03)	0.98 (0.94, 1.02)	0.98 (0.93, 1.04)	1.00 (0.92, 1.08)
OBR‐cancers	1.01 (0.96, 1.06)	1.02 (0.98, 1.07)	1.03 (0.98, 1.07)	1.01 (0.96, 1.07)	0.99 (0.95, 1.04)	0.96 (0.92, 1.00)	0.96 (0.9, 1.02)	0.97 (0.87, 1.07)
NOBR‐cancers	0.98 (0.96, 1.01)	0.98 (0.96, 1.01)	0.98 (0.95, 1.02)	0.98 (0.94, 1.03)	0.99 (0.94, 1.03)	0.99 (0.94, 1.03)	0.98 (0.93, 1.05)	1.01 (0.93, 1.11)
NOBR cancers excluding lung and prostate	0.98 (0.94, 1.01)	0.97 (0.94, 1.00)	0.96 (0.93, 1.00)	0.98 (0.91, 1.05)	0.99 (0.89, 1.10)	0.96 (0.89, 1.05)	0.94 (0.87, 1.03)	0.95 (0.85, 1.06)
Specific cancer sites								
Colorectal	1.01 (0.95, 1.09)	1.04 (0.97, 1.11)	1.04 (0.97, 1.12)	1.03 (0.97, 1.10)	1.02 (0.95, 1.08)	0.98 (0.92, 1.05)	0.97 (0.89, 1.06)	1.01 (0.89, 1.15)
Pancreas	1.02 (0.91, 1.15)	1.03 (0.92, 1.16)	1.03 (0.92, 1.15)	1.01 (0.91, 1.13)	0.99 (0.89, 1.10)	0.96 (0.87, 1.07)	0.95 (0.82, 1.10)	0.92 (0.73, 1.17)
Kidney	0.99 (0.87, 1.12)	0.98 (0.87, 1.11)	0.98 (0.87, 1.10)	0.97 (0.86, 1.09)	0.95 (0.85, 1.07)	0.93 (0.83, 1.04)	0.93 (0.80, 1.08)	0.90 (0.79, 1.03)
Lung	0.96 (0.9, 1.02)	0.93 (0.87, 0.99)	0.91 (0.85, 0.96)	0.89 (0.84, 0.95)	0.89 (0.84, 0.94)	0.89 (0.84, 0.95)	0.89 (0.84, 0.95)	0.90 (0.79, 1.03)
Prostate	0.99 (0.96, 1.03)	1.00 (0.96, 1.05)	1.01 (0.96, 1.05)	1.01 (0.97, 1.06)	1.02 (0.97, 1.08)	1.03 (0.97, 1.09)	1.04 (0.97, 1.12)	1.08 (0.97, 1.19)
Women								
All cancers	1.01 (0.99, 1.03)	1.01 (0.99, 1.03)	1.02 (0.98, 1.06)	1.03 (0.97, 1.09)	1.02 (0.96, 1.09)	1.02 (0.96, 1.09)	1.02 (0.96, 1.10)	1.00 (0.96, 1.05)
OBR‐cancers	1.02 (1.00, 1.05)	1.04 (1.01, 1.07)	1.05 (1.01, 1.09)	1.05 (1.00, 1.11)	1.05 (0.99, 1.11)	1.05 (0.99, 1.10)	1.05 (0.99, 1.11)	1.03 (0.98, 1.08)
NOBR‐cancers	0.98 (0.95, 1.02)	0.97 (0.94, 1.00)	0.96 (0.93, 0.99)	0.97 (0.92, 1.03)	0.97 (0.91, 1.03)	0.98 (0.91, 1.04)	0.98 (0.90, 1.05)	0.96 (0.92, 1.01)
NOBR cancers excluding lung	0.98 (0.95, 1.02)	0.98 (0.94, 1.02)	0.96 (0.93, 1.00)	0.98 (0.90, 1.06)	0.98 (0.90, 1.07)	0.98 (0.88, 1.10)	0.97 (0.88, 1.07)	0.94 (0.90, 0.98)
Specific cancer sites								
Colorectal	1.00 (0.94, 1.07)	1.00 (0.94, 1.06)	0.99 (0.93, 1.05)	0.98 (0.92, 1.04)	0.97 (0.92, 1.02)	0.96 (0.91, 1.02)	0.97 (0.87, 1.07)	1.01 (0.83, 1.23)
Pancreas	0.99 (0.88, 1.11)	0.98 (0.88, 1.09)	0.97 (0.87, 1.08)	0.95 (0.85, 1.05)	0.93 (0.84, 1.04)	0.93 (0.84, 1.02)	0.93 (0.84, 1.03)	0.87 (0.77, 0.98)
Kidney	1.03 (0.91, 1.15)	1.04 (0.92, 1.16)	1.03 (0.92, 1.15)	1.01 (0.90, 1.13)	0.97 (0.87, 1.08)	0.94 (0.84, 1.05)	0.91 (0.79, 1.04)	0.84 (0.71, 1.00)
Lung	0.97 (0.91, 1.03)	0.96 (0.90, 1.02)	0.95 (0.90, 1.01)	0.96 (0.90, 1.02)	0.97 (0.91, 1.02)	0.97 (0.91, 1.03)	0.99 (0.92, 1.07)	1.03 (0.93, 1.14)
Endometrial	1.03 (0.96, 1.10)	1.04 (0.98, 1.11)	1.09 (0.91, 1.31)	1.08 (0.87, 1.34)	1.04 (0.85, 1.26)	1.02 (0.84, 1.24)	0.99 (0.83, 1.18)	0.92 (0.82, 1.03)
Ovary	0.99 (0.90, 1.10)	0.99 (0.90, 1.10)	0.99 (0.90, 1.09)	0.98 (0.89, 1.07)	0.98 (0.89, 1.07)	0.98 (0.89, 1.07)	0.96 (0.88, 1.06)	0.96 (0.86, 1.07)
Postmenopausal breast	1.04 (1.00, 1.09)	1.08 (1.04, 1.13)	1.10 (1.06, 1.15)	1.12 (1.08, 1.17)	1.14 (1.09, 1.18)	1.15 (1.11, 1.19)	1.16 (1.12, 1.21)	1.18 (1.13, 1.24)

*Note*: Green—significant positive interaction. Orange—significant inverse interaction. NB: immortal time bias has been accounted for across all age periods of interest.

Abbreviations: BMI, body mass index; CI, confidence interval; HR, hazard ratio; MV, multivariable; NOBR, non‐obesity related; OBR, obesity‐related.

* Multivariable adjustment for baseline age, race, alcohol, smoking, and hormone replacement therapy (in women).

### 3.3 Sensitivity Analysis

Analysis of the BMI‐cancer associations at ages across adulthood in the ARIC cohort with multivariable adjustment including smoking as a binary measure (Table S12a) and smoking pack‐years, a cumulative measure (Table S12b), found nearly identical results, indicating that more detailed smoking information did not significantly impact the BMI–cancer associations.

Analysis was repeated on participants with ≥ 1 observed BMI measurement (Figures S5 and S6; Tables S13 and S14). HRs by age by cancer type per 5 kg/m^2^ BMI increment were similar (Figures S5 and S6). An interaction by age was found in men for obesity‐related cancers at ages 30‐unlike the main analysis with no age interactions (Table S14a). In women, for obesity‐related cancers, the sensitivity analysis found a significant interaction at age 35. Unlike main analyses, an interaction at age 30–65 between BMI and postmenopausal breast cancer was found (Table S14b).

## 4 Discussion

A higher BMI increased obesity‐related cancer risk across ages 30–65. Some evidence indicates that, relative to baseline risk, BMI–cancer associations in the fourth and fifth decades of life have a greater impact on obesity‐related cancer risk than at other ages. Generally, the limited variations in BMI‐cancer associations across adulthood suggest that adiposity at any age elevates cancer risk. Alternatively, using predicted rather than observed BMI may inadequately reflect age‐dependent variations in risk. We identified inverse interactions per 5 kg/m^2^ BMI for lung cancer in men at ages 35–60, with the inverse BMI‐cancer association strength varying by age. For women, the age interaction regarding the BMI‐cancer link was for obesity‐related cancers at ages 35 and 40 and ages 35–65 for postmenopausal breast cancer. Weight or weight trajectories before puberty were not considered, which can influence the timing of puberty or extend from early adulthood to the first AOI.

## 5 Context of Main Findings

For colorectal, pancreas, kidney, endometrial, and postmenopausal breast cancers, positive associations per 5 kg/m^2^ BMI were found at ages 40 onwards. Prior analysis of 25–49‐year‐olds found cancer incidences increased for 12 obesity‐related cancers 1995–2014, potentially from obesity prevalences [26]. We found that for colorectal cancer, the positive association for BMI was non‐significantly weaker with age. Positive associations found at age 30 and colorectal cancer risk are similar to Russo et al. (1998) [27]. Kidney cancer had positive associations per 5 kg/m^2^ BMI from age 40 in men and age 35–65 in women. An NIH‐AARP study by Deng et al. found higher BMI at ages 18, 35, 50 and baseline to be associated with a greater overall renal cancer incidence [28]; however, in our study, this was only from age 40 in men and age 35–65 in women. Another study found early and later life obesity to be associated with increased kidney cancer risk [9]. Pancreatic cancer had positive associations at ages 30–65 in men and ages 30–55 in women; however, there were no significant interactions by age. Findings may be due to residual confounding by smoking. A Norwegian study found an increased risk of pancreatic ductal adenocarcinoma per 5 kg/m^2^ exposure at ages 16–29 [29]. Li et al. found positive associations between overweight individuals across ages 14–39 and obese individuals from ages 20–49 and pancreatic cancer risk [30]. This emphasises maintaining a normal BMI across adulthood to reduce pancreatic cancer.

Age interacted with the inverse association between BMI and lung cancer, potentially influenced by smoking and residual confounding considering no adjustment for current smokers and packyears. A meta‐analysis of 29 studies showed an inverse association between BMI and lung cancer risk in never smokers [31]. Analysis by lung cancer type is necessary, given potential obesity‐cancer biological mechanisms relevant to squamous cell carcinoma and small cell lung cancers and irrelevant to adenocarcinomas [32]. Endometrial cancer findings are similar to a European study which found associations with overweight exposure before age 40 [33]. Increasing endometrial cancer incidence rates in younger populations over the last 10 years are potentially a birth cohort phenomenon rather than strictly age [26, 34]. No BMI‐ovarian cancer associations across AOIs were found, similar to a Norwegian study which only found associations in adolescence or young adulthood [35]. Early and mid‐life excess adiposity may be more relevant to ovarian cancer. Our findings regarding endometrial and ovarian cancer should be carefully interpreted given no adjustment for gestation and hysterectomies and/or oophorectomies which may influence age‐related trends, particularly given hysterectomy prevalence is higher among Black than White women in the US [36].

Understanding obesity‐cancer associations across adulthood helps generate hypotheses of underlying biological mechanisms for tailored cancer screening and prevention. Potential mechanisms associated with obesity, ageing and carcinogenesis include proinflammatory changes with age, such as increased cytokine production [37]. Other mechanisms include hyperinsulinemia, insulin resistance and raised insulin‐like growth factor‐1 [38, 39]. The consistent association observed in this study between BMI and cancer incidence across the AOIs may reflect the persistent cumulative effects of underlying biological mechanisms listed, such as inflammation, which may influence cancer development since the initiation of carcinogenesis and continue regardless of age. Consequently, the modifying effects of age may be masked by the persistent cumulative effects of underlying biological mechanisms. For postmenopausal breast cancer, the positive associations with BMI at ages 35 onwards indicate contributions to postmenopausal breast cancer development at around 10–20 years in advance of menopause [40].

This is the first SPA of BMI‐cancer associations stratified by sex and cancer types. Methods were computationally simple and scalable, unlike comparable joint‐modelling methods [14]. Multiple imputation accounted for missing covariate data. In this IPD‐metanalysis, we accounted for cohort variations, such as differential classification of tumour types, using participant‐level random effects. Ages 30–65 were analysed in this study, given the availability of data across all cohorts and AOI were defined in advance of the study as a frequent 5‐yearly interval to avoid data driven selection bias. 5‐yearly intervals were chosen to capture general age‐related trends, given our understanding of changes in BMI‐cancer associations at particular ages is limited and the purpose of the study.

There is a potential healthy survivor bias in this study, as participants were required to have survived till the AOI and be cancer‐free at that time. This selection bias may lead to an underestimation of cancer risk, particularly for BMI measured earlier in life. Another limitation is that predicted BMI values may not have considered the time‐varying nature of BMI captured with observed BMI, which may have led to an underestimation of the age‐varying effects of the BMI‐cancer link. BMI does not consider changes in body composition with ageing [41]. Modelling BMI as a continuous exposure represents an alternative analytical approach to our use of thresholding BMI and treating it as a factor variable. We chose the latter for pragmatic reasons, as it aligns with clinically meaningful thresholds. There was a low number of cancer events by cancer site, limiting analysis of less common cancers or cancer site subgroups. Another limitation is that other confounders such as physical activity, energy intake from diet, socioeconomic status (like income, education, neighbourhood socioeconomic status) could not be adjusted for given the lack of availability of variables across all cohorts and the need to harmonise data, as otherwise the validity of pooled results will be reduced and reflective of analytic choices rather than true population level differences. Cumulative exposure to smoking was not adjusted for, given the lack of data availability across all cohorts, resulting in a potential residual confounding by smoking and an underestimation of the BMI‐cancer associations. However, the sensitivity analysis of the ARIC cohort adjusting for smoking pack‐years showed little variation in the BMI‐cancer association compared with adjustment for smoking as a binary measure (ever versus never smoker). Overall, covariates were assumed to be constant, and uncertainties in covariates measured in error were not accounted for [42]. The lack of time‐updated covariates meant that covariates measured after the AOI may be a mediator rather than a confounder, so adjustment may have resulted in attenuated BMI associations, particularly for younger ages compared to older ages. This may explain why associations between BMI at younger and older ages were similar. This is important for the adjustment of HRT use, which declined in use following breast cancer and cardiovascular disease risk concerns [43]. Future work should focus on identifying datasets with time‐updated covariates and identifying whether associations remain similar across age periods or vary. If SPA is used clinically, interpretation of the conditional nature of results to a particular AOI must be provided as BMI is correlated over time [23]. We previously explored cumulative excess adiposity effects on cancer using overweight, obese‐ and waist circumference‐years [44, 45, 46]. For AOI occurring before study entry, left truncation addressed immortal time bias; however, an inherent selection bias exists as only participants who survived till study entry were included, potentially underestimating HRs of BMI‐related  ancer risks as healthier participants survived till study entry, which is a study limitation [47].

Summary estimates across more populations and ages, especially childhood, will be explored in ABACus 2 given positive associations previously found [48]. The upward shift in BMI across all ages and increased cancer incidence over time means BMI distributions across the AOI may not be generalisable to current populations [49]. Markers of biological mechanisms of the obesity‐cancer link across sensitive periods should be explored. SPA methods could identify whether obesity reversal at sensitive periods will attenuate cancer associations similar to smoking cessation. Positive associations for non‐obesity‐related cancers mean some cancers are obesity‐related beyond those defined [3].

Elevated BMI between 30 and 65 years was associated with increased obesity‐related cancer risk. This strengthens the rationale to implement targeted, thus, more effective prevention strategies at any age in adulthood to minimise BMI exposure. Variations in BMI‐cancer associations by age were limited, likely due to the lack of power and need for more regularly measured BMI and/or more studies where BMI is intervened at different ages to fully address this question.

## Author Contributions

**Nadin K. Hawwash:** funding acquisition, writing – original draft, writing – review and editing, methodology, software, formal analysis, investigation, visualisation, project administration, validation, data curation, conceptualisation. **Matthew Sperrin:** supervision, writing – review and editing, conceptualisation, validation, methodology, visualisation. **Glen P. Martin:** writing – review and editing, methodology, supervision, conceptualisation, validation, visualisation. **Rashmi Sinha:** data curation, writing – review and editing. **Charles E. Matthews:** data curation, writing – review and editing. **Matthias B. Schulze:** writing – review and editing, data curation. **Anouk Hiensch:** data curation, writing – review and editing. **Pilar Amiano:** writing – review and editing, data curation. **Marian L. Neuhouser:** data curation, writing – review and editing. **Corinne E. Joshu:** writing – review and editing, data curation. **Elizabeth A. Platz:** data curation, writing – review and editing. **Heinz Freisling:** writing – review and editing, data curation. **Marc J. Gunter:** writing – review and editing, data curation. **Andrew G. Renehan:** supervision, funding acquisition, conceptualisation, writing – review and editing, project administration, data curation.

## Funding

This work was supported by Cancer Research UK (C19941/A28707, C147/A25254), the Manchester NIHR Biomedical Research Centre (IS‐BRC‐1215‐20007), the National Cancer Institute (U01 CA164975), the National Heart, Lung, and Blood Institute (75N92022D00001, 75N92022D00002, 75N92022D00003, 75N92022D00004, 75N92022D00005, N01WH22110, 24152, 32100–2, 32105–6, 32108–9, 32111–13, 321115, 32118–32119, 32122, 42107–26, 42129–32, and 44221), National Institutes of Health, Department of Health and Human Services, U.S. Department of Health and Human Services, the Intramural Research Program of the National Cancer Institute, the International Agency for Research on Cancer, Imperial College London, European Commission (DG‐SANCO), the Danish Cancer Society, Ligue Contre le Cancer, Institut Gustave‐Roussy, Mutuelle Générale de l'Education Nationale, Institut National de la Santé et de la Recherche Médicale, Deutsche Krebshilfe, Deutsches Krebsfor‐ schungszentrum, German Federal Ministry of Education and Research, the Hellenic Health Foundation, Associa‐ zione Italiana per la Ricerca sul Cancro‐AIRC‐Italy and National Research Council, Dutch Ministry of Public Health, Welfare, and Sports, Netherlands Cancer Registry, LK Research Funds, Dutch Prevention Funds, Dutch Zorg Onderzoek Nederland, World Cancer Research Fund, Statistics Netherlands, Health Research Fund, Instituto de Salud Carlos III, regional Spanish governments of Andalucía, Asturias, Basque Country, Murcia, and Navarra, the Catalan Institute of Oncology, Swedish Cancer Society, Swedish Scientific Council, and Region Skåne and Region Västerbotten, and the Medical Research Council (MR/N003284/1 and MC‐UU_12015/1 to EPIC‐Norfolk; MR/M012190/1 to EPIC‐Oxford; UK).

## Disclosure

The funders of this study had no role in the design, data collection, data analysis and interpretation, or writing of the report. Co‐authors had full access to their cohort data and were responsible for the final decision to submit for publication.

## Ethics Statement

No ethical approval was required given the de‐identified data. Anonymous data were handled as per data management and transfer agreements. Only ARIC participants who consented to use of data more broadly than just for cardiovascular research were included. The Institutional Review Boards at each study site approved the ARIC study protocol. The NIH‐AARP Diet and Health Study was reviewed and approved by the Special Studies Institutional Review Board of the US National Cancer Institute, and all participants gave written informed consent by completing and returning the questionnaire.

## Conflicts of Interest

The authors declare no conflicts of interest.

## Supporting information


**Table S1:** Examples of prior studies that have analysed the interaction by age on the BMI‐cancer link.
**Table S2:** Datasets included in the ABACus 2 Consortium. Table reproduced from [16].
**Table S3:** PRISMA IPD Checklist.
**Table S4:** Characteristics* of the analytic cohorts. Table adapted from [16].
**Table S5:** Number of incident cancer cases identified across the follow‐up period in the ABACus2 Consortium.
**Table S6:** I2 values of the interaction by age on the BMI‐cancer link at ages 30 to 65 in men and women, ABACus 2.
**Table S7:** Hazard ratios of interaction by age on BMI‐cancer link in men and women, AARP.
**Table S8:** Hazard ratios of interaction by age on BMI‐cancer link in men and women, PLCO.
**Table S9:** Hazard ratios of interaction by age on BMI‐cancer link in men and women, ARIC.
**Table S10:** Hazard ratios of interaction by age on BMI‐cancer link in men and women, EPIC.
**Table S11:** Hazard ratios of interaction by age on BMI‐cancer link in men and women, WHI.
**Table S12:** Hazard ratios for cancer incidence per 5‐unit BMI exposure at ages 30 to 65 in men and women in the subgroup with multivariable adjustment including (A) smoking (ever/never) versus (B) smoking pack‐years, ARIC cohort.
**Table S13:** Hazard ratios for cancer incidence per 5‐unit BMI exposure at ages 30 to 65 in (A) men and (B) women in the subgroup with at least 1 BMI measurement, ABACus 2.
**Table S14:** Hazard ratio of interactions by age on the BMI‐cancer link at ages 30 to 65 in (A) men and (B) women in the subgroup with at least 1 BMI measurement, ABACus 2.
**Figure S1:** Demonstrating a hypothetical sensitive period analysis of the hazard ratio between a 5‐unit BMI exposure and the related cancer risk across adulthood.
**Figure S2:** ABACus 2 Consortium Participant flow diagram. BMI‐related exclusion criteria were observational‐level exclusions, but resulted in individual exclusions if none of the BMI readings fell within the clinically plausible range.
**Figure S3:** (A) distribution and (B) density of observed and predicted BMI for each cohort 2.
**Figure S4:** Hazard ratios for cancer incidence per 5‐unit BMI exposure at ages 30 to 65 in (A) men and (B) women in the subgroup with at least 3 BMI measurements, ABACus 2.
**Figure S5:** Hazard ratios of cancers per 5‐unit BMI (kg/m2) exposure at ages 30 to 65 for men for combined cancer subgroups and by cancer type over adulthood, ABACus 2.
**Figure S6:** Hazard ratios for cancer incidence per 5‐unit BMI (kg/m2) exposure at ages 30 to 65 for women, ABACus 2.

## Data Availability

Data are available from the cohorts after approval. Further information is available from the corresponding author upon request. ARIC: https://aric.cscc.unc.edu/aric9/, EPIC: https://epic.iarc.fr, PLCO: https://cdas.cancer.gov/datasets/plco/, WHI: https://www.whi.org/md/working‐with‐whi‐data, NIH‐AARP: https://www.nihaarpstars.com/Default.aspx?projectid=098b1a48‐4822‐4126‐8d09‐562e7d3b3659.
