# Supplementary material for: Sensitive Period Analysis of Adulthood BMI and Cancer Risk: An Individual Participant Data Meta‐Analysis of Over 720,000 Participants in the ABACus 2 Consortium
Source: Int J Cancer. 2026 Apr 8;159(4):960–72. doi: 10.1002/ijc.70464 (PMC13284623; doi:10.1002/ijc.70464)
Supplement: Supplementary file 1 — Table S1: Examples of prior studies that have analysed the interaction by age on the BMI‐cancer link. Table S2: Datasets included in the ABACus 2 Consortium. Table reproduced from [16]. Table S3: PRISMA IPD Checklist. Table S4: Characteristics* of the analytic cohorts. Table adapted from [16]. Table S5: Number of incident cancer cases identified across the follow‐up period in the ABACus2 Consortium. Table S6: I2 values of the interaction by age on the BMI‐cancer link at ages 30 to 65 in men and women, ABACus 2. Table S7: Hazard ratios of interaction by age on BMI‐cancer link in men and women, AARP. Table S8: Hazard ratios of interaction by age on BMI‐cancer link in men and women, PLCO. Table S9: Hazard ratios of interaction by age on BMI‐cancer link in men and women, ARIC. Table S10: Hazard ratios of interaction by age on BMI‐cancer link in men and women, EPIC. Table S11: Hazard ratios of interaction by age on BMI‐cancer link in men and women, WHI. Table S12: Hazard ratios for cancer incidence per 5‐unit BMI exposure at ages 30 to 65 in men and women in the subgroup with multivariable adjustment including (A) smoking (ever/never) versus (B) smoking pack‐years, ARIC cohort. Table S13: Hazard ratios for cancer incidence per 5‐unit BMI exposure at ages 30 to 65 in (A) men and (B) women in the subgroup with at least 1 BMI measurement, ABACus 2. Table S14: Hazard ratio of interactions by age on the BMI‐cancer link at ages 30 to 65 in (A) men and (B) women in the subgroup with at least 1 BMI measurement, ABACus 2. Figure S1: Demonstrating a hypothetical sensitive period analysis of the hazard ratio between a 5‐unit BMI exposure and the related cancer risk across adulthood. Figure S2: ABACus 2 Consortium Participant flow diagram. BMI‐related exclusion criteria were observational‐level exclusions, but resulted in individual exclusions if none of the BMI readings fell within the clinically plausible range. Figure S3: (A) distribution and (B) density of observed and predicted [file IJC-159-960-s001.pdf]

Sensitive period analysis of adulthood BMI and cancer risk: an individual participant data meta-analysis of over 720,000 participants in the ABACus 2 Consortium

Nadin, K, Hawwash, Matthew Sperrin, Glen, P, Martin, Rashmi, Sinha, Charles, E, Matthews, Matthias, B, Schulze, Anouk, Hiensch, Pilar, Amiano, Marian, L, Neuhouser, Corinne, E, Joshu, Elizabeth, A, Platz, Heinz, Freisling, Marc, J, Gunter, Andrew, G, Renehan

Table of contents:

- Supplementary Information

Research in context

- Supplementary tables

Table S1: Examples of prior studies that have analysed the interaction by age on the BMI-cancer link.

Table S2: Datasets included in the ABACus 2 Consortium. Table reproduced from (16).

Table S3: PRISMA IPD Checklist

Table S4: Characteristics* of the analytic cohorts. Table adapted from (16).

Table S5: Number of incident cancer cases identified across the follow-up period in the ABACus2 Consortium.

Table S6: I2 values of the interaction by age on the BMI-cancer link at ages 30 to 65 in men and women, ABACus 2.

Table S7: Hazard ratios of interaction by age on BMI-cancer link in men and women, AARP.

Table S8: Hazard ratios of interaction by age on BMI-cancer link in men and women, PLCO.

Table S9: Hazard ratios of interaction by age on BMI-cancer link in men and women, ARIC.

Table S10: Hazard ratios of interaction by age on BMI-cancer link in men and women, EPIC.

Table S11: Hazard ratios of interaction by age on BMI-cancer link in men and women, WHI.

Table S12: Hazard ratios for cancer incidence per 5-unit BMI exposure at ages 30 to 65 in men and women in the subgroup with multivariable adjustment including (A) smoking (ever/never) versus (B) smoking pack-years, ARIC cohort.

Table S13: Hazard ratios for cancer incidence per 5-unit BMI exposure at ages 30 to 65 in (A) men and (B) women in the subgroup with at least 1 BMI measurement, ABACus 2.

Table S14: Hazard ratio of interactions by age on the BMI-cancer link at ages 30 to 65 in (A) men and (B) women in the subgroup with at least 1 BMI measurement, ABACus 2.

- Supplementary figures

Figure S1: Demonstrating a hypothetical sensitive period analysis of the hazard ratio between a 5-unit BMI exposure and the related cancer risk across adulthood.

Figure S2: ABACus 2 Consortium Participant flow diagram. BMI-related exclusion criteria were observational-level exclusions, but resulted in individual exclusions if none of the BMI readings fell within the clinically plausible range.

Figure S3: (A) distribution and (B) density of observed and predicted BMI for each cohort.

Figure S4: Hazard ratios for cancer incidence per 5-unit BMI exposure at ages 30 to 65 in (A) men and (B) women in the subgroup with at least 3 BMI measurements, ABACus 2.
Figure S5: Hazard ratios of cancers per 5-unit BMI (kg/m2) exposure at ages 30 to 65 for men for combined cancer subgroups and by cancer type over adulthood, ABACus 2.
Figure S6: Hazard ratios for cancer incidence per 5-unit BMI (kg/m2) exposure at ages 30 to 65 for women, ABACus 2.

- Supplementary references

<u>Supplementary Information</u>

<u>Research in Context</u>

Evidence before this study

Excess body fatness, commonly approximated by elevated body mass index (BMI) is a major cause of cancer, being associated with at least 13 cancer types (referred to as obesity-related cancer). Globally over 500,000 or approximately 4% of all new cancers in adults are attributable to elevated BMI. To develop targeted population-level cancer prevention strategies, namely through weight loss interventions, there is a need to understand key time points during the life course where excess body fatness impacts the greatest in terms of subsequent risk of obesity-related cancers. We conducted a PubMed search to identify relevant studies published until November 2023 using the terms "BMI", "cancer", " life course", and "obesity-related cancer". Ten studies were identified; however, no study evaluated BMI associations with obesity-related cancer risk across regular age increments ('sensitive periods') and numerous cancer sites in adulthood, while accounting for immortal time bias. We assembled the ABACus 2 Consortium of five cohorts including 720,210 participants (21% were of European descendants), each with at least 3 BMI measurements collected throughout adulthood, spanning nine European countries and the United States. With a median follow-up of 9.85 years in men and 10.80 years in women. This study aimed to determine temporal increases in obesity-related cancer risks with BMI across 5-year age increments, analysed by gender.

The added value of this study

To our knowledge, this is the first study to use super-landmarking approaches to explore age-varying effects by stacking data for each participant over predicted ages of interest (AOIs) to analyse associations per 5 kg/m^2 BMI increase at the AOIs and cancer incidence, and then meta-analyse using individual participant level data. We showed that excess BMI exposure at any age between ages 30 and 65 increased the risk of cancer. However, we found no particular AOI where excess BMI-related exposures at that particular age period significantly contributed to a greater or lesser risk of cancer compared with other age increments. We found two settings of interactions of age on the BMI cancer link at ages 35 and 40 for obesity-related cancers in women and at ages 35-65 for postmenopausal breast cancer, but we cannot exclude whether these observations were significant or by chance.

Implications of all available evidence

Weight management at any age in adulthood should be encouraged worldwide, particularly in weight management trials, cancer prevention strategies, and policy changes. Prior evidence of elevated risk associated with the cumulative degree and duration of excess BMI exposure strengthens the rationale to implement effective prevention strategies early on in adulthood to minimise the obesity-related cancer risk. Studies using sensitive period analyses across the whole life course and repeated in other populations is required to inform prevention strategies for obesity-related cancers in the future.

Supplementary Tables

Table S1: Examples of prior studies that have analysed the interaction by age on the BMI-cancer link.

Study, date published	Cohort, Number of Participants included	Follow-up	BMI at age periods analysed	Main findings	Strengths	Limitations
**Adult weight change and risk of postmenopausal breast cancer** (1)**, 2006**	Nurses' Health Study, 87,143 postmenopausal women	26 years (1976-2002)	Weight change since age 18, and weight change since menopause.	Compared with women who maintained their weight, a 25kg weight gain since age 18 had a significantly higher postmenopausal cancer risk (relative risk [RR], 1.45; 95% confidence interval [CI], 1.27-1.66; P<.001 for trend) especially in never HRT users (RR,1.98; 95% CI, 1.55-2.53) and a 10kg gain in weight since menopause had an increased breast cancer risk (RR, 1.18; 95% CI, 1.03-1.35; P = .002 for trend). So weight gain in adult life prior to and after menopause increases postmenopausal breast cancer risk.	- Large cohort size.<br>- Length of follow-up.<br>- Prospective design.<br>- Biennially adjusted weight and menopause status.<br>- Alcohol was longitudinally measured and adjusted for.	- Did not account for immortal time bias.<br>- Self-reported weight and height.<br>- Limited generalisability to other populations.<br>- Limited covariate adjustment – smoking and exercise not adjusted for.
**Body mass index and risk of ovarian cancer** (2)**, 2008**	NIH-AARP Diet and Health Study, 94,525 U.S. women	7 years (1996/7-2003)	BMI at age 18 and study baseline.	BMI at baseline showed a weak positive association with ovarian cancer (RR comparing obese with normal weight women=1.14; 95%-CI=0.86–1.51)<br><br>Compared with women who were normal weight at age 18, the multivariate RRs of ovarian cancer for overweight and obese women at age 18 were 1.29 (95%-CI=0.82–2.04) and 1.74 (95% CI: 0.86–3.53), respectively.	- Large cohort size.<br>- Prospective design.	- Did not account for immortal time bias.<br>- Self-reported weight and height.<br>- Cohort mainly included Caucasian postmenopausal women so results may not apply to women of other populations.<br>- Covariates were collected at baseline only so potential misclassification over time.
**Body mass index and renal cell cancer: The influence of race and sex** (3)**, 2012**	Kidney Cancer Study, Midwest US, 2,448 men and women	Not provided	BMI at age 21, 5 years prior to interview and at interview. Maximum and minimum weight between age 21 and 2 years were reported.	Early and later life obesity were associated with increased renal cancer risk where obesity in early adulthood had an odds ratio of 1.6 (95% CI: 1.1,2.4) and obesity 5 years before diagnosis had an odds ratio of 1.6 (95% CI: 1.1, 2.2).	- Recruited large number of Black participants (56%) which allowed analysis by race.	- Did not account for immortal time bias.<br>- Self-reported weight measures were used.<br>- Smoking exposure defined at baseline and not time-updated so potential misclassification over time.<br>- Although found independent association of early and later life obesity – caution with attributing specific obesity effect to point in life course
**Association of Body Mass Index and Age with Subsequent Breast Cancer Risk in Premenopausal Women** (4)**,2018**	Premenopausal Breast Cancer Collaborative Group, 758 592 premenopausal women (19 prospective cohorts)	Median follow-up 9.3 years.	BMI at ages 18-24, 25-34, 35-44, 45 -54.	Linear inverse association for BMI at ages 18-24 HR 0.77 (95% CI: 0.73, 0.80). BMI ages 45-54 HR 0.88 (95% CI: 0.86, 0.91). BMI in early adulthood had the strongest inverse associations of premenopausal cancer risk.	- Large cohort size.<br>- All cohorts were prospective and used time-updated covariate information.<br>- Analysis by hormone receptor status at differed premenopausal ages.	- Did not account for immortal time bias.<br>- Varied hormone receptor cut points and differential classification of the tumour types between studies may have underestimated relative risks – yet fixed effects IPD-metanalysis was used given.
**Age-specific breast cancer risk by body mass index and familial risk:**	Breast Cancer Prospective Family Study Cohort (ProF-	Followed for up to 20 years (mean	BMI at baseline.	The BMI-breast cancer risk association depended on age<br>at baseline (HR = 1.05, P = 0.002).	- Adequate statistical power to examine interactions with	- Did not account for immortal time bias.<br>- BMI change with menopausal status could

**Prospective family study cohort** (5)**, 2018**	SC), 16,035 women.	10.5 years).		There was no evidence for an interaction between age at baseline and menopausal status (χ12 = 0.01).	underlying familial risk.	be unmeasured confounding.<br>- Limited power to analyse by mutation carriers and tumour hormone receptor status.
**Young adulthood body mass index, adult weight gain and breast cancer risk: the PROCAS Study (United Kingdom)** (6), **2000**	The Predicting Risk Of Cancer At Screening (PROCAS) study, 47,042 women.	Median follow-up of 5.6 years.	BMI recalled at age 20. Calculated average absolute weight gain from age 20 to cohort entry.	Weight gain in women with a BMI <23.4 kg/m2 aged 20 years increased postmenopausal breast cancer risk.	- Prospective study.<br><br>- Contemporaneous population.	- Self-reported height and weight.<br>- Mainly a Caucasian population.<br>- Residual confounding from crude classification of measures cross-sectional measures of alcohol and exercise.
**Adolescent obesity and midlife cancer risk: a population-based cohort study of 2·3 million adolescents in Israel** (7)**, 2020**	Israeli National Cancer Registry, 2.3 million – 1,370,020 men and 928,110 women.	Men: 29 542 73 5 person-years of follow-, women:18 044 863 person-years of follow-up.	BMI at age 17 years-old.	Positive associations in BMI at age 17 and cancer incidence in men and women (on exclusion of breast and ovarian cancer) with hazard ratios of 1.26 (95% CI: 1.18–1.35) and 1.27 (95% CI: 1.13–1.44) respectively.	- Large cohort size.<br><br>- Analysis by cancer types.<br><br>- No immortal time bias in this study.	- Cohort was not representative of all ethnicities so limited generalizability among other populations.<br>- No adjustment for confounders capturing the participant's lifestyle such as smoking and alcohol consumption.
**Measures of Body Fatness and Height in Early and Mid-to-Late Adulthood and Prostate Cancer Risk and Mortality in the Pooling Project of Prospective Studies of Diet and Cancer** (8), **2021**	The Pooling Project of Prospective Studies of Diet and Cancer (DCPP), 830 772 men	Varied follow-up for each prospectiv e study.	BMI between ages 18-21 and each cohort baseline.	Found no associations between BMI and metastatic prostate cancer in early adulthood but associations in mid to late adulthood (HR 1.30, 95% CI = 0.95–1.78).	- Large cohort size.<br><br>- Ancestral diversity and geographic diversity so generalizable to more populations.	- Did not account for immortal time.<br>- Covariates were collected at baseline only so potential misclassification over time.
**Associations of Body Mass Index at Different Ages With Early-Onset Colorectal Cancer** (9)**, 2022**	Darmkrebs: Chancen der Verhütung durch Screening (DACHS) study, 747 patients and 621 controls	No uniform follow-up period reported.	BMI at ages 20, 30, 10 years before diagnosis/ interview.	Compared with those with a BMI <25 kg/m2, those with BMI ≥30 kg/m2 at ages 20 and 30 years and ~10 years before diagnosis/ interview had 2.56 (95% CI, 1.20-5.44), 2.06- (95%CI, 1.25-3.40), and 1.88- (95% CI, 1.30-2.73) fold risk of early-onset CRC.	- Large case control study.	- Did not account for immortal time bias.<br>- Self-reported weight and height.<br>- No time-updated covariates were adjusted for so a potential is classification over time.
**Analysis of Body Mass Index in Early and Middle Adulthood and Estimated Risk of Gastrointestinal Cancer** (10), **2023**	Prostate, Lung, Colorectal, and Ovarian (PLCO) Cancer Screening Trial, 135 161 participants.	Mean follow-up 13.9 (6.0) years.	BMI at ages 20, 50, study entry.	Excess BMI in early (HR 1.23; 95% CI, 1.10-1.37) and middle adulthood (HR (HR, 1.23; 95% CI, 1.13-1.34) and later adulthood and later adulthood (HR, 1.21; 95% CI, 1.10-1.32) was associated with elevated colorectal cancer risk.	- Large cohort size.<br><br>- Use of observed BMI measurements.	- Did not account for immortal time.<br>- Adjusted for aspirin, ibuprofen, MI, stroke and diabetes which are potential mediators.<br>- No mention of how missing data was accounted for.<br>- Covariates were collected at baseline only so potential misclassification over time.

1. Eliassen AH, Colditz GA, Rosner B, Willett WC, Hankinson SE. Adult weight change and risk of postmenopausal breast cancer. J Am Med Assoc. 2006 Jul 12;296(2):193–201.
2. Leitzmann MF, Koebnick C, Danforth KN, Brinton LA, Moore SC, Hollenbeck AR, et al. Body mass index and risk of ovarian cancer. Cancer. 2009 Feb 2;115(4):812–22.
3. Beebe-Dimmer JL, Colt JS, Ruterbusch JJ, Keele GR, Purdue MP, Wacholder S, et al. Body mass index and renal cell cancer: The influence of race and sex. Epidemiology. 2012 Nov;23(6):821–8.
4. Schoemaker MJ, Nichols HB, Wright LB, Brook MN, Jones ME, O'Brien KM, et al. Association of Body Mass Index and Age with Subsequent Breast Cancer Risk in Premenopausal Women. JAMA Oncol. 2018 Nov 1;4(11).
5. Hopper JL, Dite GS, MacInnis RJ, Liao Y, Zeinomar N, Knight JA, et al. Age-specific breast cancer risk by body mass index and familial risk: Prospective family study cohort (ProF-SC). Breast Cancer Res. 2018 Nov 3;20(1):1–11.
6. Renehan AG, Pegington M, Harvie MN, Sperrin M, Astley SM, Brentnall AR, et al. Young adulthood body mass index, adult weight gain and breast cancer risk: the PROCAS Study (United Kingdom). Br J Cancer. 2020 May 12;122(10):1552–61.
7. Furer A, Afek A, Sommer A, Keinan-Boker L, Derazne E, Levi Z, et al. Adolescent obesity and midlife cancer risk: a population-based cohort study of 2·3 million adolescents in Israel. Lancet Diabetes Endocrinol. 2020 Mar 1;8(3):216–25.
8. Genkinger JM, Wu K, Wang M, Albanes D, Black A, van den Brandt PA, et al. Measures of body fatness and height in early and mid-to-late adulthood and prostate cancer: risk and mortality in The Pooling Project of Prospective Studies of Diet and Cancer. Ann Oncol. 2020 Jan 1;31(1):103–14.
9. Li H, Boakye D, Chen X, Jansen L, Chang-Claude J, Hoffmeister M, et al. Associations of Body Mass Index at Different Ages With Early-Onset Colorectal Cancer. Gastroenterology. 2022 Apr 1;162(4):1088–1097.e3.
10. Loomans-Kropp HA, Umar A. Analysis of Body Mass Index in Early and Middle Adulthood and Estimated Risk of Gastrointestinal Cancer. JAMA Netw open. 2023 May 10;6(5):e2310002.
11. The ARIC investigators. The atherosclerosis risk in community (ARIC) study: Design and objectives. Am J Epidemiol. 1989 Apr 1;129(4):687–702.
12. Haftenberger M, Lahmann P, Panico S, Gonzalez C, Seidell J, Boeing H, et al. Overweight, obesity and fat distribution in 50- to 64-year-old participants in the European Prospective Investigation into Cancer and Nutrition (EPIC). Public Health Nutr. 2002;5(6b):1147–62.
13. Arnold M, Jiang L, Stefanick ML, Johnson KC, Lane DS, LeBlanc ES, et al. Duration of Adulthood Overweight, Obesity, and Cancer Risk in the Women's Health Initiative: A Longitudinal Study from the United States. Prentice A, editor. PLoS Med. 2016 Aug 16;13(8):e1002081.
14. Kelly SP, Graubard BI, Andreotti G, Younes N, Cleary SD, Cook MB. Prediagnostic body mass index trajectories in relation to prostate cancer incidence and mortality in the PLCO cancer screening trial. J Natl Cancer Inst. 2017 Mar 1;109(3).
15. Klein JP, Moeschberger ML. Survival Analysis. 2003;

Table S2: Datasets included in the ABACus 2 Consortium. Table reproduced from (16).

Study	Country	Study entry date	N	BMI and WC data collection	Disease outcomes	Method of Number of cancer events follow-up
**ARIC (17)**	US	1987-1989.	15,792	Prospective weight and height collection by trained personnel using standardized protocols across centers. Weight, height and WC were collected at Visit 1-4 each 3 years apart and then 15 years later, measures were collected more frequently at Visit 5-8 [12]. BMI at age 25 was recalled.	Cancer incidence. Administrative censoring on December 31, 2015.	Linkage to 4 state cancer registries (ICD-O3 codes) supplemented with hospital discharge summaries and medical records.
**EPIC (18)**	(10 European countries*)	1990	521,000	BMI and WC data were collected at study entry and BMI at age 20 was recalled. 5 years post study entry, BMI and WC were collected by a questionnaire. Details on data collection by country have been described in-depth elsewhere [13].	Cancer Incidence. Administrative censoring was center specific (22 collaborating centres).	Registry linkage (ICD-10 cancer codes) except in Germany and France, where cancer pathology registries, active-follow-up and health insurance records were used.
**WHI (19) (Women only)**	US	1991	161,808	At baseline, recall self-reports of BMI at ages 18, 35 and 50 were collected. Height and weight measures were also collected at baseline [14].<br>Only observational data was used in this study. Clinical trial data was not used given the use of observational-level data in this IPD meta-analysis to explore BMI cancer links.	Cancer incidence. Administrative censoring for main study 2005, Extension 1 in 2010 and Extension 2 in 2020.	Annual mailed follow-up questionnaires. Cancer (ICD-10 codes).
**PLCO (20)**	US	1993	154,887	Recall BMI at ages 20 and 50 and self-reported BMI at study entry (mean age 63 years) were collected [15].	Cancer incidence. Administrative censoring in 2009.	Self-reported annual follow-up questionnaires. Cancer codes (ICD-0-3 codes).
**NIH-AARP (21)**	US	1996	566,398	Recall BMI at ages 18, 35 and 50 were collected by a questionnaire and BMI at study entry (mean age 61) was self-reported.<br>The NIH-AARP follow up questionnaire from 2004-2006 was not used in this study as only baseline covariate data was used given the lack of updated covariate data from other cohorts.	Cancer incidence. Administrative censoring in 2011.	Cancer registry linkage (ICD-0-3 codes) in the original 8 states of recruitment.

Absolute criterion: over three measures of BMI over time
* Denmark, France, Germany, Italy, the Netherlands, Norway, Spain, Sweden and the United Kingdom.
Abbreviations: N, number of participants; BMI, body mass index; WC, waist circumference; US, United States; UK, United Kingdom; EPIC, European Prospective Investigation into Cancer and Nutrition; NIH-AARP, NIH-AARP Diet and Health Study; PLCO, Prostate, Lung, Colorectal, Ovarian Cancer Screening Trial; WHI, Women's Health Initiative; ARIC, Atherosclerosis Risk in Communities study.

16.Hawwash NK, Sperrin M, Martin GP, Sinha R, Matthews CE, Ricceri F, et al. Excess weight by degree and duration and cancer risk (ABACus2 consortium): a cohort study and individual participant data meta-analysis. eClinicalMedicine. 2024 Dec 1;78.
17. Joshu CE, Barber JR, Coresh J, Couper DJ, Mosley TH, Vitolins MZ, et al. Enhancing the infrastructure of the atherosclerosis risk in Communities (ARIC) study for cancer epidemiology research: Aric cancer. Cancer Epidemiol Biomarkers Prev. 2018 Mar 1;27(3):295–305.
18. Riboli E, Kaaks R. The EPIC Project: Rationale and study design. Int J Epidemiol. 1997;26(SUPPL. 1).
19. Anderson G, Cummings S, Freedman LS, Furberg C, Henderson M, Johnson SR, et al. Design of the Women's Health Initiative clinical trial and observational study. Control Clin Trials. 1998;19(1):61–109.
20. Black A, Huang W-Y, Wright P, Riley T, Mabie J, Mathew S, et al. PLCO: Evolution of an Epidemiologic Resource and Opportunities for Future Studies. Rev Recent Clin Trials. 2015 Oct 7;10(3):238–45.
21. Schatzkin A, Subar AF, Thompson FE, Harlan LC, Tangrea J, Hollenbeck AR, et al. Design and serendipity in establishing a large cohort with wide dietary intake distributions: The National Institutes of Health-American Association of Retired Persons Diet and Health Study. Am J Epidemiol. 2001 Dec 15;154(12):1119–25

Table S3: PRISMA IPD Checklist

PRISMA-IPD Section/topic	Item No	Checklist item	Reported on page
**Title**			
Title	1	Identify the report as a systematic review and meta-analysis of individual participant data.	1
**Abstract**			
Structured summary	2	Provide a structured summary including as applicable:	2
		**Background**: state research question and main objectives, with information on participants, interventions, comparators and outcomes.	
		**Methods**: report eligibility criteria; data sources including dates of last bibliographic search or elicitation, noting that IPD were sought; methods of assessing risk of bias.	
		**Results**: provide number and type of studies and participants identified and number (%) obtained; summary effect estimates for main outcomes (benefits and harms) with confidence intervals and measures of statistical heterogeneity. Describe the direction and size of summary effects in terms meaningful to those who would put findings into practice.	
		**Discussion:** state main strengths and limitations of the evidence, general interpretation of the results and any important implications.	
		**Other:** report primary funding source, registration number and registry name for the systematic review and IPD meta-analysis.	
**Introduction**			
Rationale	3	Describe the rationale for the review in the context of what is already known.	3
Objectives	4	Provide an explicit statement of the questions being addressed with reference, as applicable, to participants, interventions, comparisons, outcomes and study design (PICOS). Include any hypotheses that relate to particular types of participant-level subgroups.	3
**Methods**			
Protocol and registration	5	Indicate if a protocol exists and where it can be accessed. If available, provide registration information including registration number and registry name. Provide publication details, if applicable.	
Eligibility criteria	6	Specify inclusion and exclusion criteria including those relating to participants, interventions, comparisons, outcomes, study design and characteristics (e.g. years when conducted, required minimum follow-up). Note whether these were applied at the study or individual level i.e. whether eligible participants were included (and ineligible participants excluded) from a study that included a wider population than specified by the review inclusion criteria. The rationale for criteria should be stated.	4
Identifying studies - information sources	7	Describe all methods of identifying published and unpublished studies including, as applicable: which bibliographic databases were searched with dates of coverage; details of any hand searching including of conference proceedings; use of study registers and agency or company databases; contact with the original research team and experts in the field; open adverts and surveys. Give the date of last search or elicitation.	4
Identifying studies - search	8	Present the full electronic search strategy for at least one database, including any limits used, such that it could be repeated.	NA
Study selection processes	9	State the process for determining which studies were eligible for inclusion.	NA

Data collection processes	10	Describe how IPD were requested, collected and managed, including any processes for querying and confirming data with investigators. If IPD were not sought from any eligible study, the reason for this should be stated (for each such study).<br><br>If applicable, describe how any studies for which IPD were not available were dealt with. This should include whether, how and what aggregate data were sought or extracted from study reports and publications (such as extracting data independently in duplicate) and any processes for obtaining and confirming these data with investigators.	Data coverage was based on availability so risk of bias assessment was not applicable.
Data items	11	Describe how the information and variables to be collected were chosen. List and define all study level and participant level data that were sought, including baseline and follow-up information. If applicable, describe methods of standardising or translating variables within the IPD datasets to ensure common scales or measurements across studies.	5
IPD integrity	A1	Describe what aspects of IPD were subject to data checking (such as sequence generation, data consistency and completeness, baseline imbalance) and how this was done.	NA
Risk of bias assessment in individual studies.	12	Describe methods used to assess risk of bias in the individual studies and whether this was applied separately for each outcome. If applicable, describe how findings of IPD checking were used to inform the assessment. Report if and how risk of bias assessment was used in any data synthesis.	Data coverage was based on availability so risk of bias assessment was not applicable.
Specification of outcomes and effect measures	13	State all treatment comparisons of interests. State all outcomes addressed and define them in detail. State whether they were pre-specified for the review and, if applicable, whether they were primary/main or secondary/additional outcomes. Give the principal measures of effect (such as risk ratio, hazard ratio, difference in means) used for each outcome.	4-7
Synthesis methods	14	Describe the meta-analysis methods used to synthesise IPD. Specify any statistical methods and models used. Issues should include (but are not restricted to):<br>• Use of a one-stage or two-stage approach.<br>• How effect estimates were generated separately within each study and combined across studies (where applicable).<br>• Specification of one-stage models (where applicable) including how clustering of patients within studies was accounted for.<br>• Use of fixed or random effects models and any other model assumptions, such as proportional hazards.<br>• How (summary) survival curves were generated (where applicable).<br>• Methods for quantifying statistical heterogeneity (such as I^2 and τ^2).<br>• How studies providing IPD and not providing IPD were analysed together (where applicable).<br>• How missing data within the IPD were dealt with (where applicable).	
Exploration of variation in effects	A2	If applicable, describe any methods used to explore variation in effects by study or participant level characteristics (such as estimation of interactions between effect and covariates). State all participant-level characteristics that were analysed as potential effect modifiers, and whether these were pre-specified.	
Risk of bias across studies	15	Specify any assessment of risk of bias relating to the accumulated body of evidence, including any pertaining to not obtaining IPD for particular studies, outcomes or other variables.	Data coverage was based on availability so risk of bias assessment was not

			applicable.
Additional analyses	16	Describe methods of any additional analyses, including sensitivity analyses. State which of these were pre-specified.	
**Results**			
Study selection and IPD obtained	17	Give numbers of studies screened, assessed for eligibility, and included in the systematic review with reasons for exclusions at each stage. Indicate the number of studies and participants for which IPD were sought and for which IPD were obtained. For those studies where IPD were not available, give the numbers of studies and participants for which aggregate data were available. Report reasons for non-availability of IPD. Include a flow diagram.	
Study characteristics	18	For each study, present information on key study and participant characteristics (such as description of interventions, numbers of participants, demographic data, unavailability of outcomes, funding source, and if applicable duration of follow-up). Provide (main) citations for each study. Where applicable, also report similar study characteristics for any studies not providing IPD.	6-7
IPD integrity	A3	Report any important issues identified in checking IPD or state that there were none.	None.
Risk of bias within studies	19	Present data on risk of bias assessments. If applicable, describe whether data checking led to the up-weighting or down-weighting of these assessments. Consider how any potential bias impacts on the robustness of meta-analysis conclusions.	Data coverage was based on availability so risk of bias assessment was not applicable.
Results of individual studies	20	For each comparison and for each main outcome (benefit or harm), for each individual study report the number of eligible participants for which data were obtained and show simple summary data for each intervention group (including, where applicable, the number of events), effect estimates and confidence intervals. These may be tabulated or included on a forest plot.	NA – would not be possible to present given the number of cancer types, exposures and cohorts included
Results of syntheses	21	Present summary effects for each meta-analysis undertaken, including confidence intervals and measures of statistical heterogeneity. State whether the analysis was pre-specified, and report the numbers of studies and participants and, where applicable, the number of events on which it is based.	6-7
		When exploring variation in effects due to patient or study characteristics, present summary interaction estimates for each characteristic examined, including confidence intervals and measures of statistical heterogeneity. State whether the analysis was pre-specified. State whether any interaction is consistent across trials.	
		Provide a description of the direction and size of effect in terms meaningful to those who would put findings into practice.	
Risk of bias across studies	22	Present results of any assessment of risk of bias relating to the accumulated body of evidence, including any pertaining to the availability and representativeness of available studies, outcomes or other variables.	Data coverage was based on availability so risk of bias assessment was not applicable.

Additional analyses	23	Give results of any additional analyses (e.g. sensitivity analyses). If applicable, this should also include any analyses that incorporate aggregate data for studies that do not have IPD. If applicable, summarise the main meta-analysis results following the inclusion or exclusion of studies for which IPD were not available.	
**Discussion**			
Summary of evidence	24	Summarise the main findings, including the strength of evidence for each main outcome.	**8**
Strengths and limitations	25	Discuss any important strengths and limitations of the evidence including the benefits of access to IPD and any limitations arising from IPD that were not available.	**10**
Conclusions	26	Provide a general interpretation of the findings in the context of other evidence.	**11**
Implications	A4	Consider relevance to key groups (such as policy makers, service providers and service users). Consider implications for future research.	**11**
**Funding**			
Funding	27	Describe sources of funding and other support (such as supply of IPD), and the role in the systematic review of those providing such support.	**11-12**

Table S4: Characteristics* of the analytic cohorts. Table adapted from (16).

Characteristic	Men (N = 312,123)				Women (N = 408,078)				
	**ARIC N = 5,897**	**PLCO N = 71,667**	**NIH-AARP N = 187,528**	**EPIC N = 47,040**	**ARIC N = 7,566**	**PLCO N = 74,087**	**NIH-AARP N = 126,906**	**EPIC N = 108,486**	**WHI N = 91,033**
**Age *, years, mean (SD)**	57.0 (6.0)	63.0 (5.0)	62.0(5.3)	60.0 (8.0)	57.0(6.0)	63.0 (5.0)	62.0 (5.0)	57.0(10.0)	64.0(7.0)
**Height*, meters, mean (SD)**	1.76(0.07)	1.78(0.08)	1.78 (0.07)	1.76 (0.07)	1.62(0.06)	1.63(0.02)	1.63 (0.06)	1.64 (0.06)	1.62 (0.07)
**Measured BMI *, kg/m^2 mean (SD)**	27.7 (4.3)	27.5 (4.2)	27.2 (4.2)	26.3 (3.5)	28.2 (6.1)	27.1 (5.5)	26.8 (5.8)	25.3 (4.2)	27.2 (5.8)
**Ethnicity, n (%)**									
**White**	4,707 (80)	63,452 (89)	176,095(95)		5,475 (72)	65,856 (89)	116,400(93)		76,213(84)
**Black**	1,190 (20)	3,163(4)	3,961(2)	NA	2,091 (28)	4,076 (6)	5,582 (5)	NA	10,287(11)
**Other**	NA	5,012 (7)	5,670 (3)		NA	4,129 (6)	3,443 (3)		4,281 (5)
**Missing**	0 (0)	40 (0)	0 (0)		0 (0)	26 (0)	0 (0)		252 (0)
**Smoking, n (%)**									
**Ever**	4,349 (74)	45,514(64)	125,537(69)	31,224(67)	3,745 (50)	32,856(44)	67,396 (55)	50,190(50)	44,146(49)
**Never**	1,533 (26)	26,141 (37)	55,489 (31)	15,357(33)	3,810 (50)	41,226(56)	55,789 (45)	50,293(50)	42,927(47)
**Missing**	15 (0)	12 (0)	6,502 (3)	459 (1)	11 (0)	5 (0)	3,721 (3)	8,003 (7)	1,219 (1)
**Alcohol consumption, units of alcohol per week*, mean (SD)**	4 (9)	9 (26)	8 (20)	15 (19)	1 (4)	9 (26)	3 (8)	7 (9)	2 (5)
**Missing**	20 (0)	16,463 (22)	0 (0)	20,688(43)	11 (0)	16,463(22)	0 (0)	31,496(29)	47 (0)
**HRT, n (%)**									
**Ever**					4,109 (54)	49,058(67)	77,513 (62)	25,884(28)	54,296(60)
**Never**					2,242 (35)	24,578(33)	47,797 (38)	66,873(72)	36,659(40)
**Missing**					1,215 (16)	356 (0)	1,596 (1)	15,729 (14)	78 (0)

Values in parentheses are percentages unless otherwise stated.
*These values are based on the index date.
Abbreviations: N, number of participants; BMI, body mass index; HRT, hormone replacement therapy; US, United States; UK, United Kingdom; EPIC, European Prospective Investigation into Cancer and Nutrition[1]; NIH-AARP, Diet and Health Study; PLCO, Prostate, Lung, Colorectal, Ovarian Cancer Screening Trial; WHI, Women's Health Initiative; ARIC, Atherosclerosis Risk in Communities study; NA, not applicable.
[1]Denmark, France, Germany, Italy, the Netherlands, Norway, Spain, Sweden and the United Kingdom.

16.Hawwash NK, Sperrin M, Martin GP, Sinha R, Matthews CE, Ricceri F, et al. Excess weight by degree and duration and cancer risk (ABACus2 consortium): a cohort study and individual participant data meta-analysis. eClinicalMedicine. 2024 Dec 1;78.

Table S5: Number of incident cancer cases identified across the follow-up period in the ABACus2 Consortium.

Cancer type	Number of men diagnosed with cancer	Number of women diagnosed with cancer
†Total Cancers	85,341	63,732
OBR-cancers	12,959	36,509
NOBR-cancers	64,743	24,499
NOBR-cancers excluding lung and prostate	26,178	16,352
**Specific cancer sites**		
Colorectal	6,037	6,251
Pancreas	1,957	2,019
Kidney	1,967	1,270
Lung	8,559	8,114
Prostate	30,006	-
Endometrial	-	3,931
Ovarian	-	2,717
Post-menopausal breast cancer	-	17,582

† The sum of OBR and NOBR cancer does not equal total cancers as non-melanoma skin cancers were excluded in the EPIC cohort analyses.
Abbreviations: OBR, obesity-related; NOBR, non-obesity related

Table S6: I^2 values of the interaction by age on the BMI-cancer link at ages 30 to 65 in men and women, ABACus 2.

Outcomes	I^2							
	Men							
	Age 30	Age 35	Age 40	Age 45	Age 50	Age 55	Age 60	Age 65
**All Cancers**	0.00	0.00	0.36	0.60	0.54	0.68	0.86	0.92
**OBR-cancers**	0.00	0.00	0.00	0.30	0.00	0.00	0.05	0.59
**NOBR-cancers**	0.00	0.00	0.23	0.53	0.60	0.70	0.83	0.90
**NOBR cancers excluding lung and prostate**	0.00	0.00	0.00	0.43	0.65	0.55	0.58	0.67
**Specific cancer sites**								
**Colorectal**	0.00	0.00	0.16	0.16	0.00	0.00	0.25	0.48
**Pancreas**	0.00	0.00	0.00	0.00	0.00	0.00	0.00	0.31
**Kidney**	0.00	0.00	0.00	0.00	0.00	0.00	0.39	0.00
**Lung**	0.00	0.00	0.00	0.00	0.31	0.00	0.18	0.49
**Prostate**	0.00	0.00	0.00	0.21	0.47	0.51	0.72	0.84
**Women**								
**All Cancers**	0.00	0.12	0.68	0.82	0.84	0.84	0.85	0.69
**OBR-cancers**	0.00	0.00	0.50	0.69	0.72	0.69	0.73	0.62
**NOBR-cancers**	0.00	0.00	0.06	0.56	0.61	0.67	0.70	0.25
**NOBR cancers excluding lung**	0.00	0.00	0.19	0.60	0.65	0.75	0.67	0.00
**Specific cancer sites**								
**Colorectal**	0.00	0.00	0.00	0.00	0.31	0.12	0.60	0.79
**Pancreas**	0.00	0.00	0.00	0.00	0.00	0.00	0.00	0.00
**Kidney**	0.00	0.00	0.00	0.20	0.14	0.04	0.31	0.34
**Lung**	0.00	0.00	0.00	0.00	0.00	0.00	0.24	0.44
**Endometrial**	0.00	0.39	0.77	0.84	0.82	0.82	0.75	0.55
**Ovary**	0.00	0.00	0.00	0.00	0.00	0.00	0.00	0.00
**Postmenopausal breast**	0.00	0.00	0.00	0.00	0.00	0.00	0.00	0.00

Abbreviations: OBR, obesity-related; NOBR, non-obesity related; I^2, heterogeneity.

Table S7: Hazard ratios of interaction by age on BMI-cancer link in men and women, AARP.

Outcomes	MV-adjusted HR (95% CI)							
	Men							
	Age 30	Age 35	Age 40	Age 45	Age 50	Age 55	Age 60	Age 65
All Cancers	0.98 (0.96,1)	0.98 (0.96,1)	0.97 (0.95,0.99)	0.96 (0.94,0.98)	0.96 (0.94,0.98)	0.94 (0.92,0.96)	0.93 (0.91,0.95)	0.91 (0.89,0.93)
OBR-cancers	1 (0.94,1.06)	1.01 (0.96,1.07)	1.01 (0.96,1.07)	0.99 (0.94,1.04)	0.98 (0.93,1.03)	0.95 (0.9,1)	0.92 (0.87,0.97)	0.89 (0.84,0.94)
NOBR-cancers	0.97 (0.94,1)	0.97 (0.94,1)	0.96 (0.93,0.99)	0.95 (0.93,0.97)	0.95 (0.93,0.97)	0.94 (0.92,0.96)	0.93 (0.91,0.95)	0.92 (0.89,0.95)
NOBR cancers excluding lung and prostate	0.97 (0.93,1.01)	0.96 (0.92,1)	0.95 (0.91,0.99)	0.94 (0.9,0.98)	0.93 (0.9,0.97)	0.91 (0.88,0.94)	0.89 (0.86,0.92)	0.87 (0.83,0.91)
Specific cancer sites								
Colorectal	1 (0.92,1.09)	1.02 (0.94,1.11)	1.02 (0.94,1.11)	1.01 (0.93,1.09)	1 (0.93,1.08)	0.98 (0.91,1.06)	0.93 (0.86,1.01)	0.92 (0.84,1)
Pancreas	0.98 (0.84,1.14)	0.97 (0.84,1.12)	0.96 (0.83,1.11)	0.94 (0.82,1.08)	0.93 (0.81,1.06)	0.9 (0.79,1.03)	0.87 (0.76,1)	0.89 (0.76,1.04)
Kidney	1.01 (0.88,1.16)	1.02 (0.89,1.17)	1.01 (0.89,1.15)	0.99 (0.87,1.12)	0.97 (0.86,1.1)	0.94 (0.83,1.06)	0.89 (0.78,1.01)	0.8 (0.69,0.93)
Lung	0.95 (0.88,1.03)	0.93 (0.86,1)	0.91 (0.84,0.98)	0.9 (0.84,0.97)	0.9 (0.84,0.97)	0.9 (0.84,0.97)	0.89 (0.83,0.96)	0.87 (0.8,0.94)
Prostate	0.98 (0.94,1.02)	0.98 (0.94,1.02)	0.98 (0.94,1.02)	0.98 (0.94,1.02)	0.98 (0.94,1.02)	0.98 (0.94,1.02)	0.98 (0.94,1.02)	0.97 (0.93,1.01)
Women								
All Cancers	1 (0.97,1.03)	1 (0.97,1.03)	0.99 (0.96,1.02)	0.99 (0.96,1.02)	0.99 (0.96,1.02)	0.97 (0.94,1)	0.96 (0.93,0.99)	0.96 (0.93,0.99)
OBR-cancers	1.02 (0.98,1.06)	1.03 (0.99,1.07)	1.03 (0.99,1.07)	1.02 (0.98,1.06)	1.02 (0.98,1.06)	1.01 (0.97,1.05)	1 (0.96,1.04)	0.99 (0.95,1.03)
NOBR-cancers	0.97 (0.92,1.02)	0.95 (0.9,1)	0.94 (0.9,0.99)	0.93 (0.89,0.97)	0.93 (0.89,0.97)	0.92 (0.88,0.96)	0.91 (0.87,0.95)	0.92 (0.87,0.97)
NOBR cancers excluding lung	0.97 (0.91,1.03)	0.96 (0.9,1.02)	0.94 (0.89,1)	0.93 (0.88,0.98)	0.93 (0.88,0.98)	0.91 (0.86,0.96)	0.91 (0.86,0.96)	0.92 (0.87,0.98)
Specific cancer sites								
Colorectal	0.99 (0.89,1.1)	0.98 (0.88,1.09)	0.97 (0.88,1.07)	0.95 (0.86,1.04)	0.94 (0.86,1.03)	0.93 (0.85,1.02)	0.89 (0.81,0.98)	0.88 (0.79,0.97)
Pancreas	0.96 (0.8,1.15)	0.94 (0.79,1.12)	0.92 (0.78,1.09)	0.9 (0.77,1.06)	0.89 (0.76,1.04)	0.9 (0.77,1.05)	0.9 (0.77,1.06)	0.83 (0.7,0.99)
Kidney	1 (0.82,1.22)	0.99 (0.82,1.2)	0.97 (0.81,1.17)	0.94 (0.79,1.12)	0.92 (0.77,1.09)	0.88 (0.74,1.05)	0.83 (0.69,1)	0.77 (0.63,0.95)
Lung	0.95 (0.86,1.04)	0.93 (0.85,1.02)	0.92 (0.84,1)	0.92 (0.85,1)	0.93 (0.86,1.01)	0.92 (0.85,1)	0.92 (0.85,1)	0.93 (0.85,1.02)
Endometrial	1.01 (0.91,1.12)	1 (0.91,1.1)	0.96 (0.88,1.05)	0.92 (0.84,1.01)	0.89 (0.82,0.97)	0.88 (0.81,0.96)	0.87 (0.79,0.95)	0.85 (0.76,0.95)
Ovary	0.98 (0.82,1.17)	0.97 (0.82,1.15)	0.97 (0.82,1.15)	0.96 (0.82,1.13)	0.97 (0.83,1.13)	0.95 (0.81,1.11)	0.93 (0.79,1.09)	0.98 (0.82,1.17)
Postmenopausal breast	1.05 (0.99,1.11)	1.09 (1.03,1.15)	1.11 (1.05,1.17)	1.13 (1.07,1.19)	1.14 (1.08,1.2)	1.15 (1.09,1.21)	1.15 (1.09,1.21)	1.16 (1.09,1.23)

* Multivariable adjustment for baseline age, race, alcohol, smoking and hormone replacement therapy (in women).
Green – significant positive interaction. Orange – significant inverse interaction.
NB: immortal time bias has been accounted for across all age periods of interest.
Abbreviations: OBR, obesity-related; NOBR, non-obesity related; CI, confidence interval; HR, hazard ratio; BMI, body mass index; MV, multivariable.

Table S8: Hazard ratios of interaction by age on BMI-cancer link in men and women, PLCO.

Outcomes	MV-adjusted HR (95% CI)							
	Men							
	Age 30	Age 35	Age 40	Age 45	Age 50	Age 55	Age 60	Age 65
All Cancers	1.01 (0.94,1.09)	1.02 (0.94,1.11)	1.04 (0.95,1.13)	1.05 (0.96,1.14)	1.05 (0.96,1.14)	1.01 (0.92,1.11)	1 (0.9,1.11)	1.05 (0.91,1.22)
OBR-cancers	1.05 (0.89,1.24)	1.11 (0.93,1.32)	1.18 (0.98,1.42)	1.2 (1,1.44)	1.12 (0.93,1.35)	0.96 (0.78,1.18)	1 (0.79,1.26)	1.13 (0.82,1.56)
NOBR-cancers	1 (0.92,1.09)	1 (0.91,1.1)	1 (0.91,1.1)	1.01 (0.92,1.11)	1.03 (0.94,1.13)	1.02 (0.92,1.13)	1 (0.89,1.13)	1.03 (0.87,1.22)
NOBR cancers excluding lung and prostate	1.01 (0.87,1.17)	1.03 (0.88,1.21)	1.08 (0.91,1.28)	1.14 (0.96,1.35)	1.19 (1.01,1.4)	1.13 (0.95,1.35)	1.11 (0.9,1.37)	1.11 (0.83,1.48)
Specific cancer sites								
Colorectal	1.09 (0.87,1.37)	1.21 (0.95,1.55)	1.31 (1.01,1.7)	1.28 (0.99,1.65)	1.14 (0.88,1.47)	0.89 (0.67,1.18)	0.84 (0.6,1.18)	0.9 (0.6,1.36)
Pancreas	1.01 (0.63,1.62)	1.06 (0.64,1.75)	1.17 (0.7,1.96)	1.3 (0.78,2.16)	1.28 (0.78,2.11)	1.33 (0.77,2.29)	1.64 (0.87,3.08)	1.38 (0.37,5.13)
Kidney	1 (0.65,1.54)	1.02 (0.64,1.63)	1.05 (0.64,1.71)	1.09 (0.67,1.77)	0.98 (0.59,1.63)	0.79 (0.45,1.39)	0.88 (0.46,1.69)	0.85 (0.29,2.47)
Lung	0.96 (0.79,1.17)	0.9 (0.72,1.12)	0.81 (0.64,1.02)	0.75 (0.59,0.95)	0.71 (0.56,0.89)	0.76 (0.6,0.96)	0.77 (0.59,1.01)	0.81 (0.56,1.18)
Prostate	1 (0.88,1.13)	1.02 (0.89,1.17)	1.03 (0.89,1.19)	1.04 (0.9,1.2)	1.07 (0.93,1.23)	1.07 (0.92,1.24)	1.04 (0.88,1.23)	1.07 (0.84,1.36)
Women								
All Cancers	1.03 (0.96,1.1)	1.08 (1.01,1.16)	1.13 (1.05,1.21)	1.17 (1.09,1.25)	1.17 (1.09,1.25)	1.17 (1.09,1.26)	1.19 (1.09,1.29)	1.16 (1.02,1.31)
OBR-cancers	1.04 (0.96,1.13)	1.11 (1.02,1.21)	1.17 (1.07,1.28)	1.19 (1.09,1.29)	1.19 (1.1,1.29)	1.19 (1.09,1.3)	1.21 (1.09,1.34)	1.22 (1.05,1.42)
NOBR-cancers	1 (0.89,1.12)	1.02 (0.91,1.15)	1.06 (0.94,1.2)	1.11 (0.99,1.25)	1.12 (1,1.25)	1.14 (1.01,1.29)	1.17 (1.02,1.34)	1.09 (0.89,1.34)
NOBR cancers excluding lung	1.01 (0.88,1.16)	1.06 (0.92,1.23)	1.12 (0.97,1.3)	1.18 (1.03,1.36)	1.19 (1.04,1.36)	1.24 (1.07,1.43)	1.23 (1.04,1.45)	1.13 (0.88,1.46)
Specific cancer sites								
Colorectal	1.02 (0.82,1.26)	1.06 (0.85,1.33)	1.12 (0.89,1.41)	1.16 (0.93,1.44)	1.22 (0.99,1.5)	1.15 (0.91,1.45)	1.26 (0.98,1.62)	1.6 (1.16,2.2)
Pancreas	1.06 (0.76,1.48)	1.11 (0.78,1.59)	1.1 (0.76,1.59)	1.03 (0.72,1.47)	0.98 (0.69,1.38)	0.88 (0.61,1.27)	1.12 (0.76,1.65)	1.16 (0.63,2.13)
Kidney	1.1 (0.78,1.56)	1.24 (0.86,1.78)	1.34 (0.93,1.93)	1.34 (0.95,1.88)	1.18 (0.84,1.67)	1.14 (0.8,1.63)	1.19 (0.78,1.82)	0.98 (0.53,1.82)
Lung	0.97 (0.8,1.18)	0.95 (0.77,1.17)	0.95 (0.77,1.18)	0.96 (0.78,1.18)	0.99 (0.81,1.22)	0.95 (0.76,1.18)	1.06 (0.83,1.36)	1.07 (0.76,1.52)
Endometrial	1.17 (0.9,1.52)	1.45 (1.11,1.9)	1.72 (1.32,2.24)	1.77 (1.39,2.26)	1.62 (1.27,2.07)	1.61 (1.24,2.1)	1.6 (1.14,2.24)	1.43 (0.85,2.41)
Ovary	1.04 (0.73,1.47)	1.09 (0.75,1.58)	1.14 (0.78,1.66)	1.14 (0.8,1.63)	1.08 (0.76,1.54)	1.09 (0.74,1.62)	0.97 (0.58,1.62)	1.1 (0.52,2.33)
Postmenopausal breast	1.02 (0.9,1.15)	1.05 (0.92,1.2)	1.09 (0.96,1.24)	1.12 (0.99,1.27)	1.15 (1.02,1.3)	1.19 (1.05,1.35)	1.18 (1.01,1.37)	1.1 (0.87,1.39)

* Multivariable adjustment for baseline age, race, alcohol, smoking and hormone replacement therapy (in women).
Green – significant positive interaction. Orange – significant inverse interaction.
NB: immortal time bias has been accounted for across all age periods of interest.
Abbreviations: OBR, obesity-related; NOBR, non-obesity related; CI, confidence interval; HR, hazard ratio; BMI, body mass index; MV, multivariable.

Table S9: Hazard ratios of interaction by age on BMI-cancer link in men and women, ARIC.

Outcomes	MV-adjusted HR (95% CI)							
	Men							
	Age 30	Age 35	Age 40	Age 45	Age 50	Age 55	Age 60	Age 65
All Cancers	1.01 (0.93,1.1)	1.01 (0.93,1.1)	1.01 (0.93,1.09)	1 (0.93,1.08)	1 (0.93,1.08)	1 (0.93,1.08)	0.98 (0.91,1.06)	1 (0.92,1.09)
OBR-cancers	1.04 (0.87,1.24)	1.06 (0.89,1.26)	1.07 (0.91,1.26)	1.06 (0.9,1.25)	1.05 (0.9,1.23)	1.04 (0.89,1.21)	1.02 (0.87,1.2)	0.98 (0.82,1.17)
NOBR-cancers	1 (0.89,1.12)	1 (0.89,1.12)	1.01 (0.9,1.13)	1.01 (0.91,1.12)	1.01 (0.91,1.12)	1.02 (0.92,1.13)	0.99 (0.89,1.1)	1.06 (0.94,1.19)
NOBR cancers excluding lung and prostate	1.01 (0.73,1.39)	1.02 (0.75,1.39)	1.02 (0.75,1.38)	1.02 (0.76,1.37)	0.99 (0.74,1.32)	0.99 (0.75,1.31)	0.9 (0.67,1.2)	1.08 (0.79,1.47)
Specific cancer sites								
Colorectal	1.05 (0.83,1.33)	1.09 (0.86,1.37)	1.1 (0.88,1.38)	1.11 (0.89,1.38)	1.11 (0.9,1.37)	1.1 (0.89,1.35)	1.13 (0.91,1.4)	1.13 (0.9,1.43)
Pancreas	1.01 (0.62,1.64)	1 (0.63,1.6)	0.98 (0.62,1.54)	0.96 (0.62,1.49)	0.93 (0.6,1.43)	0.94 (0.62,1.43)	0.79 (0.51,1.23)	0.71 (0.43,1.17)
Kidney	1.07 (0.63,1.81)	1.11 (0.67,1.85)	1.11 (0.68,1.82)	1.1 (0.68,1.78)	1.1 (0.69,1.76)	1.12 (0.71,1.77)	1.02 (0.63,1.66)	0.97 (0.57,1.66)
Lung	0.93 (0.68,1.27)	0.88 (0.65,1.19)	0.86 (0.64,1.15)	0.85 (0.64,1.13)	0.85 (0.64,1.12)	0.85 (0.65,1.12)	0.77 (0.58,1.02)	0.78 (0.58,1.05)
Prostate	1.01 (0.88,1.16)	1.03 (0.9,1.18)	1.04 (0.91,1.18)	1.04 (0.92,1.18)	1.05 (0.93,1.19)	1.07 (0.95,1.21)	1.06 (0.94,1.2)	1.14 (0.99,1.31)
Women								
All Cancers	1.01 (0.95,1.08)	1.01 (0.95,1.07)	1.01 (0.95,1.07)	1 (0.94,1.06)	1 (0.95,1.06)	1 (0.95,1.06)	1 (0.94,1.06)	1.01 (0.95,1.08)
OBR-cancers	1.03 (0.95,1.11)	1.04 (0.97,1.12)	1.05 (0.98,1.13)	1.05 (0.98,1.13)	1.04 (0.97,1.11)	1.05 (0.98,1.13)	1.04 (0.97,1.12)	1.04 (0.96,1.13)
NOBR-cancers	0.95 (0.77,1.17)	0.91 (0.74,1.12)	0.9 (0.74,1.1)	0.88 (0.73,1.07)	0.89 (0.74,1.07)	0.91 (0.76,1.09)	0.91 (0.75,1.1)	0.96 (0.78,1.19)
NOBR cancers excluding lung	0.96 (0.67,1.39)	0.93 (0.65,1.33)	0.91 (0.65,1.28)	0.87 (0.62,1.22)	0.89 (0.64,1.23)	0.92 (0.67,1.27)	0.86 (0.61,1.2)	0.86 (0.6,1.24)
Specific cancer sites								
Colorectal	1.01 (0.83,1.23)	1.01 (0.83,1.22)	1.01 (0.84,1.22)	1 (0.84,1.2)	0.99 (0.83,1.18)	1.03 (0.87,1.23)	1.03 (0.86,1.23)	1.05 (0.87,1.27)
Pancreas	0.99 (0.68,1.44)	0.97 (0.67,1.4)	0.96 (0.67,1.37)	0.95 (0.68,1.34)	0.93 (0.67,1.3)	0.9 (0.65,1.25)	0.81 (0.57,1.14)	0.76 (0.52,1.12)
Kidney	0.95 (0.58,1.55)	0.92 (0.58,1.47)	0.89 (0.57,1.4)	0.85 (0.55,1.32)	0.75 (0.48,1.16)	0.78 (0.51,1.2)	0.69 (0.43,1.1)	0.56 (0.32,0.98)
Lung	0.94 (0.73,1.22)	0.9 (0.7,1.16)	0.89 (0.7,1.13)	0.88 (0.7,1.11)	0.88 (0.7,1.11)	0.91 (0.73,1.14)	0.93 (0.74,1.18)	1.01 (0.78,1.31)
Endometrial	1.01 (0.8,1.27)	1 (0.8,1.25)	0.96 (0.78,1.19)	0.93 (0.76,1.14)	0.89 (0.73,1.09)	0.85 (0.69,1.04)	0.82 (0.66,1.02)	0.76 (0.59,0.99)
Ovary	0.99 (0.75,1.31)	0.99 (0.75,1.3)	0.97 (0.75,1.26)	0.95 (0.74,1.23)	0.92 (0.71,1.18)	0.93 (0.72,1.2)	0.89 (0.68,1.16)	0.92 (0.68,1.24)
Postmenopausal breast	1.05 (0.93,1.18)	1.09 (0.97,1.22)	1.12 (1.01,1.25)	1.15 (1.04,1.28)	1.17 (1.06,1.29)	1.18 (1.07,1.31)	1.19 (1.07,1.33)	1.23 (1.09,1.39)

* Multivariable adjustment for baseline age, race, alcohol, smoking and hormone replacement therapy (in women).
Green – significant positive interaction. Orange – significant inverse interaction.
NB: immortal time bias has been accounted for across all age periods of interest.
Abbreviations: OBR, obesity-related; NOBR, non-obesity related; CI, confidence interval; HR, hazard ratio; BMI, body mass index; MV, multivariable.

Table S10: Hazard ratios of interaction by age on BMI-cancer link in men and women, EPIC.

Outcomes	MV-adjusted HR (95% CI)							
	Men							
	Age 30	Age 35	Age 40	Age 45	Age 50	Age 55	Age 60	Age 65
All Cancers	1.01 (0.96,1.06)	1.01 (0.96,1.06)	1.01 (0.97,1.06)	1.01 (0.97,1.06)	1 (0.96,1.04)	1 (0.96,1.04)	1.04 (1,1.09)	1.07 (1.02,1.12)
OBR-cancers	1.02 (0.91,1.15)	1.03 (0.92,1.15)	1.02 (0.91,1.14)	1 (0.9,1.11)	0.99 (0.89,1.1)	0.97 (0.88,1.07)	1 (0.9,1.11)	1.04 (0.92,1.17)
NOBR-cancers	1.01 (0.96,1.06)	1.01 (0.96,1.06)	1.01 (0.96,1.06)	1.01 (0.96,1.06)	1.01 (0.96,1.06)	1.01 (0.96,1.06)	1.04 (0.99,1.09)	1.08 (1.02,1.14)
NOBR cancers excluding lung and prostate	1 (0.91,1.09)	0.99 (0.91,1.08)	0.98 (0.9,1.07)	0.97 (0.89,1.05)	0.96 (0.89,1.04)	0.96 (0.89,1.04)	0.97 (0.89,1.05)	0.98 (0.89,1.07)
Specific cancer sites								
Colorectal	1.01 (0.84,1.21)	1.02 (0.86,1.21)	1.01 (0.85,1.2)	1 (0.85,1.18)	0.99 (0.84,1.16)	0.97 (0.83,1.13)	1.02 (0.87,1.2)	1.12 (0.94,1.34)
Pancreas	1.01 (0.74,1.38)	1.01 (0.75,1.37)	1 (0.74,1.34)	0.99 (0.74,1.32)	0.98 (0.74,1.3)	0.97 (0.74,1.28)	1.03 (0.78,1.37)	1.01 (0.74,1.38)
Kidney	1.06 (0.79,1.43)	1.08 (0.81,1.44)	1.09 (0.82,1.44)	1.08 (0.82,1.42)	1.07 (0.82,1.39)	1.06 (0.82,1.37)	1.11 (0.85,1.45)	1.14 (0.84,1.55)
Lung	0.98 (0.84,1.14)	0.96 (0.83,1.11)	0.94 (0.81,1.08)	0.93 (0.81,1.07)	0.93 (0.81,1.07)	0.93 (0.81,1.06)	0.97 (0.85,1.11)	1.05 (0.9,1.22)
Prostate	1.02 (0.95,1.09)	1.04 (0.97,1.11)	1.04 (0.97,1.11)	1.05 (0.98,1.12)	1.06 (0.99,1.13)	1.06 (1,1.13)	1.11 (1.04,1.18)	1.16 (1.08,1.25)
Women								
All Cancers	1 (0.96,1.04)	1 (0.96,1.04)	0.99 (0.95,1.03)	0.98 (0.94,1.02)	0.97 (0.93,1.01)	0.98 (0.94,1.02)	0.98 (0.94,1.02)	0.97 (0.93,1.01)
OBR-cancers	1.01 (0.95,1.07)	1.02 (0.96,1.08)	1.01 (0.95,1.07)	1 (0.94,1.06)	0.99 (0.94,1.05)	1 (0.95,1.06)	0.99 (0.93,1.05)	0.97 (0.91,1.04)
NOBR-cancers	0.99 (0.94,1.05)	0.98 (0.93,1.04)	0.97 (0.92,1.02)	0.96 (0.91,1.01)	0.95 (0.9,1)	0.96 (0.91,1.01)	0.96 (0.91,1.01)	0.97 (0.91,1.03)
NOBR cancers excluding lung	0.99 (0.93,1.06)	0.98 (0.92,1.04)	0.96 (0.9,1.02)	0.95 (0.89,1.01)	0.94 (0.89,1)	0.94 (0.89,1)	0.95 (0.89,1.01)	0.95 (0.89,1.02)
Specific cancer sites								
Colorectal	1.01 (0.91,1.12)	1.01 (0.91,1.12)	1 (0.9,1.11)	0.99 (0.9,1.09)	0.97 (0.88,1.07)	0.98 (0.89,1.08)	0.99 (0.9,1.09)	1.02 (0.92,1.13)
Pancreas	1 (0.81,1.24)	1 (0.81,1.23)	1 (0.82,1.22)	0.99 (0.81,1.21)	0.99 (0.82,1.2)	0.98 (0.81,1.19)	0.97 (0.79,1.18)	0.88 (0.7,1.1)
Kidney	1.06 (0.86,1.31)	1.09 (0.89,1.34)	1.1 (0.9,1.34)	1.09 (0.9,1.32)	1.08 (0.89,1.3)	1.05 (0.87,1.27)	1.04 (0.85,1.27)	1.03 (0.82,1.29)
Lung	1 (0.89,1.13)	1 (0.89,1.12)	1 (0.89,1.12)	1.01 (0.91,1.13)	1.01 (0.91,1.12)	1.02 (0.92,1.14)	1.03 (0.92,1.15)	1.06 (0.94,1.19)
Endometrial	1.03 (0.91,1.17)	1.05 (0.93,1.19)	1.05 (0.93,1.18)	1.04 (0.93,1.17)	1.03 (0.92,1.15)	1.05 (0.94,1.18)	1.02 (0.9,1.15)	1.02 (0.89,1.17)
Ovary	0.98 (0.83,1.16)	0.97 (0.82,1.14)	0.95 (0.81,1.12)	0.94 (0.8,1.1)	0.94 (0.81,1.1)	0.95 (0.81,1.11)	0.96 (0.82,1.13)	0.92 (0.76,1.11)
Postmenopausal breast	1.02 (0.47,2.22)	1.02 (0.48,2.18)	1.01 (0.48,2.12)	0.98 (0.48,2.01)	0.97 (0.48,1.96)	1.1 (0.55,2.2)	1.21 (0.6,2.43)	1.02 (0.42,2.45)

* Multivariable adjustment for baseline age, race, alcohol, smoking and hormone replacement therapy (in women).
Green – significant positive interaction. Orange – significant inverse interaction.
NB: immortal time bias has been accounted for across all age periods of interest.
Abbreviations: OBR, obesity-related; NOBR, non-obesity related; CI, confidence interval; HR, hazard ratio; BMI, body mass index; MV, multivariable.

Table S11: Hazard ratios of interaction by age on BMI-cancer link in men and women, WHI.

Outcomes	MV-adjusted HR (95% CI)							
	Men							
	Age 30	Age 35	Age 40	Age 45	Age 50	Age 55	Age 60	Age 65
Women								
All Cancers	1.02 (0.97,1.08)	1.03 (0.98,1.09)	1.03 (0.98,1.08)	1.03 (0.98,1.08)	1.02 (0.97,1.07)	1.02 (0.97,1.07)	1.04 (0.99,1.09)	1.03 (0.98,1.09)
OBR-cancers	1.03 (0.96,1.1)	1.04 (0.98,1.11)	1.04 (0.98,1.11)	1.04 (0.98,1.1)	1.04 (0.98,1.1)	1.03 (0.97,1.09)	1.07 (1.01,1.13)	1.06 (0.99,1.13)
NOBR-cancers	1 (0.91,1.1)	0.99 (0.9,1.09)	0.99 (0.91,1.08)	0.99 (0.91,1.08)	0.99 (0.91,1.08)	0.99 (0.91,1.07)	0.99 (0.91,1.08)	1.01 (0.92,1.11)
NOBR cancers excluding lung	1 (0.89,1.12)	0.99 (0.89,1.1)	0.98 (0.88,1.09)	0.97 (0.88,1.07)	0.97 (0.88,1.07)	0.96 (0.87,1.06)	0.96 (0.87,1.06)	0.95 (0.85,1.06)
Specific cancer sites								
Colorectal	0.99 (0.83,1.18)	0.97 (0.82,1.15)	0.95 (0.8,1.12)	0.93 (0.79,1.09)	0.91 (0.78,1.06)	0.89 (0.76,1.04)	0.85 (0.72,1)	0.8 (0.67,0.95)
Pancreas	0.99 (0.71,1.38)	0.97 (0.7,1.34)	0.96 (0.71,1.31)	0.95 (0.7,1.28)	0.94 (0.7,1.26)	0.94 (0.71,1.25)	0.93 (0.69,1.25)	1.01 (0.74,1.38)
Kidney	1 (0.75,1.33)	0.98 (0.75,1.29)	0.95 (0.73,1.24)	0.92 (0.71,1.19)	0.89 (0.69,1.14)	0.86 (0.67,1.1)	0.86 (0.66,1.11)	0.81 (0.6,1.09)
Lung	1 (0.83,1.2)	1 (0.84,1.19)	1.01 (0.85,1.19)	1.03 (0.88,1.21)	1.04 (0.89,1.22)	1.05 (0.9,1.22)	1.09 (0.93,1.28)	1.18 (1,1.39)
Endometrial	1.04 (0.87,1.24)	1.04 (0.88,1.23)	1.03 (0.88,1.21)	1 (0.86,1.17)	0.98 (0.84,1.14)	0.95 (0.82,1.1)	0.98 (0.84,1.14)	0.92 (0.77,1.1)
Ovary	1.04 (0.78,1.38)	1.07 (0.82,1.4)	1.09 (0.84,1.42)	1.1 (0.85,1.42)	1.11 (0.87,1.42)	1.11 (0.87,1.41)	1.13 (0.88,1.45)	1.01 (0.77,1.33)
Postmenopausal breast	1.04 (0.95,1.14)	1.07 (0.98,1.17)	1.08 (0.99,1.17)	1.1 (1.02,1.19)	1.1 (1.02,1.19)	1.11 (1.03,1.2)	1.18 (1.09,1.28)	1.22 (1.12,1.33)

* Multivariable adjustment for baseline age, race, alcohol, smoking and hormone replacement therapy (in women).
Green – significant positive interaction. Orange – significant inverse interaction.
NB: immortal time bias has been accounted for across all age periods of interest.
Abbreviations: OBR, obesity-related; NOBR, non-obesity related; CI, confidence interval; HR, hazard ratio; BMI, body mass index; MV, multivariable.

Table S12: Hazard ratios for cancer incidence per 5-unit BMI exposure at ages 30 to 65 in men and women in the subgroup with multivariable adjustment including (A) smoking (ever/never) versus (B) smoking pack-years, ARIC cohort.

A)

Outcomes	MV-adjusted HR (95% CI)							
	Men							
	**Age 30**	**Age 35**	**Age 40**	**Age 45**	**Age 50**	**Age 55**	**Age 60**	**Age 65**
All Cancers	1.02 (0.97,1.08)	1.04 (0.97,1.11)	1.05 (0.98,1.13)	1.06 (0.98,1.13)	1.05 (0.98,1.12)	1.01 (0.94,1.09)	1.01 (0.92,1.1)	1.05 (0.91,1.20)
OBR-cancers	1.16 (1.02,1.31)	1.23 (1.06,1.41)	1.29 (1.11,1.5)	1.30 (1.12,1.51)	1.21 (1.04,1.40)	1.03 (0.87,1.23)	1.09 (0.88,1.34)	1.2 (0.88,1.64)
NOBR-cancers	0.99 (0.93,1.06)	0.99 (0.92,1.07)	0.99 (0.92,1.08)	1.00 (0.92,1.08)	1.01 (0.94,1.09)	1.01 (0.92,1.10)	0.99 (0.89,1.1)	1.01 (0.87,1.18)
NOBR cancers excluding lung and prostate	0.95 (0.85,1.06)	0.97 (0.85,1.1)	1.01 (0.88,1.15)	1.05 (0.91,1.2)	1.09 (0.96,1.25)	1.05 (0.9,1.21)	1.04 (0.87,1.25)	1.04 (0.79,1.36)
Specific cancer sites								
Colorectal	1.45 (1.22,1.72)	1.62 (1.33,1.97)	1.76 (1.42,2.19)	1.73 (1.4,2.14)	1.53 (1.24,1.89)	1.19 (0.93,1.52)	1.14 (0.84,1.54)	1.15 (0.77,1.71)
Pancreas	0.85 (0.59,1.21)	0.88 (0.59,1.31)	0.95 (0.62,1.45)	1.04 (0.7,1.56)	1.03 (0.7,1.51)	1.07 (0.69,1.65)	1.34 (0.78,2.3)	1.12 (0.31,4.02)
Kidney	0.96 (0.69,1.33)	0.96 (0.66,1.4)	0.98 (0.65,1.47)	1 (0.68,1.48)	0.9 (0.59,1.36)	0.72 (0.45,1.16)	0.8 (0.45,1.43)	0.74 (0.26,2.09)
Lung	1.01 (0.87,1.18)	0.95 (0.79,1.13)	0.86 (0.71,1.04)	0.79 (0.65,0.96)	0.74 (0.61,0.9)	0.79 (0.65,0.97)	0.8 (0.63,1.02)	0.84 (0.59,1.19)
Prostate	1.01 (0.92,1.11)	1.03 (0.92,1.14)	1.04 (0.93,1.17)	1.05 (0.94,1.18)	1.07 (0.96,1.2)	1.07 (0.95,1.22)	1.04 (0.89,1.2)	1.07 (0.85,1.33)
Women								
All Cancers	1.00 (0.95,1.06)	1.05 (0.99,1.11)	1.13 (1.06,1.19)	1.13 (1.07,1.19)	1.13 (1.07,1.19)	1.13 (1.07,1.20)	1.14 (1.06,1.23)	1.14 (1.01,1.29)
OBR-cancers	1.05 (0.99,1.12)	1.12 (1.04,1.20)	1.20 (1.11,1.28)	1.20 (1.12,1.28)	1.19 (1.12,1.27)	1.19 (1.11,1.28)	1.19 (1.08,1.31)	1.26 (1.09,1.47)
NOBR-cancers	0.92 (0.85,1.01)	0.94 (0.85,1.03)	1.00 (0.91,1.11)	1.00 (0.91,1.10)	1.02 (0.94,1.12)	1.04 (0.94,1.14)	1.07 (0.94,1.20)	0.98 (0.80,1.20)
NOBR cancers excluding lung	0.93 (0.84,1.04)	0.97 (0.86,1.08)	1.06 (0.95,1.20)	1.06 (0.95,1.19)	1.07 (0.97,1.19)	1.12 (1.00,1.26)	1.13 (0.98,1.30)	1.02 (0.08,1.31)
Specific cancer sites								
Colorectal	0.98 (0.83,1.15)	1.02 (0.85,1.21)	1.09 (0.91,1.31)	1.09 (0.92,1.29)	1.16 (0.99,1.36)	1.08 (0.89,1.30)	1.16 (0.93,1.44)	1.60 (1.17,2.18)
Pancreas	1.40 (1.08,1.81)	1.46 (1.09,1.95)	1.31 (0.97,1.78)	1.31 (0.99,1.75)	1.22 (0.93,1.61)	1.09 (0.80,1.49)	1.39 (0.99,1.95)	1.24 (0.64,2.39)
Kidney	1.23 (0.95,1.60)	1.37 (1.02,1.82)	1.45 (1.09,1.94)	1.45 (1.11,1.89)	1.36 (1.03,1.79)	1.30 (0.98,1.73)	1.40 (0.96,2.04)	1.17 (0.64,2.14)
Lung	0.91 (0.78,1.06)	0.89 (0.75,1.05)	0.88 (0.74,1.05)	0.88 (0.75,1.04)	0.92 (0.78,1.08)	0.88 (0.73,1.05)	0.94 (0.76,1.18)	0.94 (0.68,1.32)
Endometrial	1.21 (1.00,1.47)	1.49 (1.21,1.84)	1.80 (1.47,2.21)	1.80 (1.51,2.15)	1.65 (1.38,1.97)	1.66 (1.35,2.04)	1.61 (1.19,2.19)	1.49 (0.88,2.52)
Ovary	1.09 (0.84,1.43)	1.14 (0.85,1.54)	1.17 (0.86,1.58)	1.17 (0.88,1.54)	1.10 (0.84,1.45)	1.15 (0.83,1.60)	0.99 (0.62,1.60)	0.98 (0.46,2.10)
Postmenopausal breast	1.00 (0.91,1.09)	1.03 (0.93,1.14)	1.09 (0.98,1.21)	1.09 (0.99,1.21)	1.12 (1.02,1.23)	1.16 (1.05,1.29)	1.12 (0.98,1.29)	1.14 (0.91,1.43)

Multivariable adjustment for baseline age, race, alcohol, smoking* and hormone replacement therapy (in women).
*Smoking quantified as a binary outcome (ever or never smoker).
NB: immortal time bias has been accounted for across all age periods of interest.
Abbreviations: OBR, obesity-related; NOBR, non-obesity related; CI, confidence interval; HR, hazard ratio; BMI, body mass index; MV, multivariable.

B)

Outcomes	MV-adjusted HR (95% CI)							
	Men							
	**Age 30**	**Age 35**	**Age 40**	**Age 45**	**Age 50**	**Age 55**	**Age 60**	**Age 65**
All Cancers	1.02 (0.97,1.09)	1.04 (0.97,1.11)	1.05 (0.98,1.13)	1.06 (0.98,1.13)	1.05 (0.98,1.12)	1.01 (0.93,1.09)	1.00 (0.91,1.10)	1.04 (0.91,1.20)
OBR-cancers	1.16 (1.03,1.32)	1.23 (1.07,1.42)	1.30 (1.11,1.51)	1.30 (1.12,1.51)	1.21 (1.04,1.40)	1.04 (0.87,1.23)	1.09 (0.88,1.34)	1.20 (0.88,1.64)
NOBR-cancers	0.99 (0.93,1.06)	0.99 (0.92,1.07)	0.99 (0.92,1.08)	1.00 (0.92,1.08)	1.01 (0.93,1.09)	1.00 (0.92,1.10)	0.98 (0.89,1.09)	1.01 (0.86,1.18)
NOBR cancers excluding lung and prostate	0.95 (0.85,1.07)	0.97 (0.85,1.10)	1.00 (0.87,1.15)	1.04 (0.91,1.19)	1.08 (0.95,1.23)	1.04 (0.90,1.20)	1.02 (0.85,1.23)	1.02 (0.78,1.34)
Specific cancer sites								
Colorectal	1.45 (1.23,1.72)	1.62 (1.33,1.97)	1.76 (1.42,2.19)	1.73 (1.4,2.14)	1.53 (1.24,1.89)	1.19 (0.93,1.52)	1.14 (0.84,1.55)	1.15 (0.77,1.71)
Pancreas	0.84 (0.59,1.21)	0.87 (0.58,1.31)	0.94 (0.62,1.44)	1.03 (0.69,1.55)	1.02 (0.69,1.50)	1.06 (0.68,1.64)	1.34 (0.78,2.31)	1.1 (0.31,3.97)
Kidney	0.96 (0.69,1.34)	0.97 (0.66,1.42)	0.99 (0.66,1.48)	1.01 (0.68,1.49)	0.91 (0.6,1.37)	0.73 (0.45,1.18)	0.81 (0.46,1.44)	0.76 (0.27,2.13)
Lung	1.00 (0.85,1.16)	0.93 (0.78,1.11)	0.84 (0.69,1.03)	0.78 (0.64,0.95)	0.72 (0.6,0.88)	0.76 (0.62,0.94)	0.78 (0.61,1.00)	0.82 (0.57,1.19)
Prostate	1.02 (0.93,1.12)	1.03 (0.93,1.15)	1.05 (0.93,1.18)	1.06 (0.94,1.19)	1.07 (0.96,1.2)	1.08 (0.95,1.23)	1.04 (0.89,1.21)	1.06 (0.85,1.33)
Women								
All Cancers	1.01 (0.96,1.06)	1.05 (0.99,1.11)	1.13 (1.07,1.20)	1.13 (1.07,1.19)	1.14 (1.08,1.20)	1.14 (1.08,1.21)	1.14 (1.06,1.23)	1.12 (0.99,1.26)
OBR-cancers	1.06 (0.99,1.13)	1.12 (1.05,1.20)	1.20 (1.12,1.29)	1.20 (1.13,1.29)	1.20 (1.13,1.28)	1.20 (1.12,1.29)	1.19 (1.09,1.31)	1.25 (1.07,1.46)
NOBR-cancers	0.93 (0.85,1.01)	0.94 (0.86,1.04)	1.00 (0.91,1.11)	1.00 (0.92,1.10)	1.02 (0.94,1.12)	1.04 (0.94,1.15)	1.07 (0.94,1.21)	0.94 (0.77,1.15)
NOBR cancers excluding lung	0.93 (0.83,1.03)	0.96 (0.86,1.08)	1.07 (0.95,1.2)	1.07 (0.95,1.19)	1.07 (0.97,1.19)	1.12 (1.00,1.26)	1.14 (0.99,1.32)	1.03 (0.80,1.31)
Specific cancer sites								
Colorectal	0.99 (0.84,1.17)	1.03 (0.86,1.23)	1.10 (0.92,1.32)	1.10 (0.93,1.30)	1.16 (0.99,1.36)	1.08 (0.89,1.31)	1.18 (0.95,1.46)	1.59 (1.16,2.17)
Pancreas	1.43 (1.11,1.85)	1.50 (1.12,2.01)	1.35 (1.00,1.84)	1.35 (1.01,1.81)	1.25 (0.95,1.66)	1.13 (0.82,1.55)	1.38 (0.99,1.93)	1.15 (0.59,2.25)
Kidney	1.23 (0.94,1.60)	1.36 (1.02,1.81)	1.44 (1.08,1.93)	1.44 (1.11,1.88)	1.35 (1.02,1.78)	1.30 (0.98,1.72)	1.39 (0.95,2.03)	1.16 (0.64,2.11)
Lung	0.97 (0.84,1.12)	0.94 (0.79,1.11)	0.89 (0.74,1.06)	0.89 (0.75,1.06)	0.90 (0.76,1.07)	0.85 (0.71,1.03)	0.90 (0.71,1.14)	0.76 (0.53,1.10)
Endometrial	1.19 (0.97,1.46)	1.48 (1.20,1.83)	1.81 (1.47,2.23)	1.81 (1.51,2.17)	1.66 (1.39,1.99)	1.68 (1.36,2.07)	1.65 (1.22,2.24)	1.67 (1.00,2.78)
Ovary	1.10 (0.84,1.43)	1.15 (0.85,1.54)	1.18 (0.87,1.59)	1.18 (0.89,1.55)	1.11 (0.85,1.46)	1.15 (0.83,1.60)	1.00 (0.62,1.60)	0.98 (0.46,2.09)
Postmenopausal breast	1.00 (0.91,1.10)	1.03 (0.93,1.15)	1.10 (0.98,1.22)	1.10 (0.99,1.21)	1.12 (1.02,1.23)	1.16 (1.05,1.29)	1.12 (0.98,1.28)	1.10 (0.88,1.38)

Multivariable adjustment for baseline age, race, alcohol, packyears and hormone replacement therapy (in women).
* Smoking quantified as a cumulative measure with pack-years (the number of packs of cigarettes smoked per day multiplied by the number of years the person has smoked).
NB: immortal time bias has been accounted for across all age periods of interest.
Abbreviations: OBR, obesity-related; NOBR, non-obesity related; CI, confidence interval; HR, hazard ratio; BMI, body mass index; MV, multivariable.

Sensitivity Analysis with BMI predicted in the subgroup with at least 1 BMI measurement

Table S13: Hazard ratios for cancer incidence per 5-unit BMI exposure at ages 30 to 65 in (A) men and (B) women in the subgroup with at least 1 BMI measurement, ABACus 2.

(A)

	MV-adjusted HR (95% CI) per 5 unit BMI (kg/m^2)															
	Men															
	Age 30	I^2	Age 35	I^2	Age 40	I^2	Age 45	I^2	Age 50	I^2	Age 55	I^2	Age 60	I^2	Age 65	I^2
All Cancers	1.05 (0.95 ,1.17)	0.96	1.06 (0.95 ,1.19)	0.97	1.06 (0.95 ,1.19)	0.97	1.06 (0.96 ,1.17)	0.97	1.06 (0.95 ,1.17)	0.98	1.04 (0.95 ,1.15)	0.97	1.04 (0.98 ,1.11)	0.90	1.06 (1.04 ,1.08)	0.00
OBR-cancers	1.36 (1.14 ,1.64)	0.84	1.41 (1.20 ,1.65)	0.85	1.42 (1.25 ,1.61)	0.84	1.40 (1.27 ,1.56)	0.80	1.36 (1.21 ,1.53)	0.84	1.30 (1.09 ,1.54)	0.85	1.32 (1.27 ,1.38)	0.30	1.28 (1.24 ,1.32)	0.00
NOBR-cancers	0.97 (0.86 ,1.10)	0.96	0.98 (0.86 ,1.11)	0.96	0.98 (0.88 ,1.1)	0.96	0.98 (0.89 ,1.09)	0.96	0.99 (0.89 ,1.09)	0.96	0.99 (0.90 ,1.09)	0.97	0.99 (0.92 ,1.06)	0.91	1.01 (0.99 ,1.04)	0.00
NOBR cancers excluding lung and prostate	1.11 (0.93 ,1.33)	0.89	1.12 (0.95 ,1.32)	0.89	1.13 (1.00 ,1.29)	0.87	1.13 (1.02 ,1.25)	0.86	1.13 (1.03 ,1.23)	0.86	1.11 (1.02 ,1.21)	0.83	1.11 (1.06 ,1.16)	0.18	1.10 (1.08 ,1.12)	0.00
Specific cancer sites																
Colorectal	1.35 (1.22 ,1.49)	0.30	1.38 (1.16 ,1.64)	0.69	1.39 (1.12 ,1.73)	0.77	1.38 (1.13 ,1.68)	0.76	1.32 (1.14 ,1.53)	0.73	1.26 (1.14 ,1.41)	0.69	1.26 (1.22 ,1.31)	0.00	1.25 (1.21 ,1.29)	0.00
Pancreas	1.45 (0.91 ,2.31)	0.81	1.49 (0.95 ,2.34)	0.80	1.51 (1.02 ,2.26)	0.77	1.51 (1.08 ,2.11)	0.73	1.45 (1 ,2.12)	0.75	1.39 (0.84 ,2.28)	0.78	1.46 (1.29 ,1.65)	0.42	1.39 (1.22 ,1.57)	0.00
Kidney	1.23 (0.85 ,1.77)	0.68	1.26 (0.92 ,1.74)	0.64	1.29 (1 ,1.66)	0.58	1.29 (1.07 ,1.56)	0.44	1.26 (1.06 ,1.51)	0.44	1.25 (1.06 ,1.46)	0.37	1.22 (1.17 ,1.28)	0.00	1.20 (1.15 ,1.24)	0.00
Lung	0.89 (0.67 ,1.19)	0.92	0.86 (0.65 ,1.13)	0.92	0.82 (0.64 ,1.06)	0.93	0.80 (0.64 ,1.02)	0.93	0.79 (0.61 ,1.02)	0.95	0.81 (0.65 ,1.00)	0.93	0.79 (0.61 ,1.03)	0.92	0.83 (0.65 ,1.06)	0.82
Prostate	0.92 (0.83 ,1.02)	0.77	0.93 (0.84 ,1.03)	0.79	0.94 (0.85 ,1.04)	0.77	0.95 (0.86 ,1.06)	0.84	0.97 (0.87 ,1.08)	0.86	0.98 (0.86 ,1.10)	0.90	0.97 (0.91 ,1.04)	0.60	1.00 (0.90 ,1.12)	0.70

* Multivariable adjustment for baseline age, race, alcohol, smoking and hormone replacement therapy (in women).
Green – significant positive interaction. Orange – significant inverse interaction.
Abbreviations: OBR, obesity-related; NOBR , non-obesity related; CI, confidence interval; HR, hazard ratio; BMI, body mass index; MV, multivariable.

(B)

	MV-adjusted HR (95% CI) per 5 unit BMI (kg/m^2)															
	Women															
	**Age 30**	**I^2**	**Age 35**	**I^2**	**Age 40**	**I^2**	**Age 45**	**I^2**	**Age 50**	**I^2**	**Age 55**	**I^2**	**Age 60**	**I^2**	**Age 65**	**I^2**
**All Cancers**	1.09 (1.02 ,1.16)	0.86	1.10 (1.05 ,1.15)	0.87	1.11 (1.07 ,1.14)	0.84	1.10 (1.07 ,1.14)	0.81	1.10 (1.07 ,1.13)	0.78	1.09 (1.07 ,1.12)	0.65	1.09 (1.07 ,1.11)	0.30	1.08 (1.07 ,1.09)	0.00
**OBR-cancers**	1.16 (1.07 ,1.26)	0.88	1.18 (1.11 ,1.26)	0.85	1.19 (1.14 ,1.24)	0.82	1.19 (1.14 ,1.23)	0.74	1.18 (1.14 ,1.22)	0.73	1.17 (1.14 ,1.20)	0.57	1.16 (1.15 ,1.18)	0.00	1.15 (1.13 ,1.17)	0.00
**NOBR-cancers**	0.94 (0.80 ,1.10)	0.84	0.94 (0.80 ,1.11)	0.86	0.94 (0.80 ,1.11)	0.86	0.95 (0.79 ,1.14)	0.89	0.94 (0.78 ,1.14)	0.90	0.96 (0.81 ,1.13)	0.88	0.97 (0.82 ,1.15)	0.86	0.98 (0.89 ,1.09)	0.54
**NOBR cancers excluding lung**	0.99 (0.80 ,1.21)	0.81	1.00 (0.83 ,1.20)	0.76	1.01 (0.85 ,1.19)	0.72	1.01 (0.83 ,1.23)	0.77	1.01 (0.84 ,1.21)	0.77	1.03 (0.87 ,1.22)	0.74	1.03 (0.89 ,1.19)	0.64	1.04 (0.97 ,1.12)	0.24
**Specific cancer sites**																
**Colorectal**	1.18 (1.04 ,1.35)	0.72	1.19 (1.07 ,1.32)	0.70	1.18 (1.10 ,1.27)	0.59	1.17 (1.10 ,1.24)	0.57	1.15 (1.09 ,1.21)	0.53	1.14 (1.10 ,1.18)	0.00	1.13 (1.10 ,1.15)	0.00	1.10 (1.03 ,1.17)	0.43
**Pancreas**	1.15 (1.06 ,1.25)	0.00	1.14 (1.05 ,1.24)	0.00	1.12 (1.04 ,1.2)	0.00	1.10 (1.04 ,1.17)	0.00	1.09 (1.04 ,1.14)	0.00	1.08 (1.04 ,1.11)	0.00	1.06 (0.98 ,1.15)	0.27	1.04 (0.97 ,1.12)	0.00
**Kidney**	1.60 (1.43 ,1.79)	0.26	1.61 (1.51 ,1.71)	0.00	1.57 (1.52 ,1.63)	0.00	1.52 (1.48 ,1.56)	0.00	1.46 (1.39 ,1.54)	0.00	1.40 (1.35 ,1.46)	0.00	1.35 (1.28 ,1.43)	0.00	1.29 (1.22 ,1.36)	0.00
**Lung**	0.87 (0.79 ,0.97)	0.72	0.86 (0.79 ,0.94)	0.55	0.85 (0.80 ,0.91)	0.41	0.85 (0.80 ,0.91)	0.43	0.86 (0.80 ,0.93)	0.55	0.87 (0.81 ,0.92)	0.47	0.90 (0.86 ,0.93)	0.00	0.92 (0.90 ,0.93)	0.00
**Endometrial**	1.62 (1.27 ,2.06)	0.93	1.68 (1.43 ,1.99)	0.93	1.69 (1.48 ,1.93)	0.92	1.64 (1.45 ,1.86)	0.91	1.58 (1.42 ,1.77)	0.92	1.54 (1.40 ,1.69)	0.86	1.52 (1.37 ,1.68)	0.84	1.44 (1.31 ,1.59)	0.68
**Ovary**	1.06 (0.93 ,1.20)	0.45	1.06 (0.94 ,1.20)	0.52	1.06 (0.94 ,1.20)	0.57	1.05 (0.94 ,1.17)	0.54	1.04 (0.95 ,1.15)	0.51	1.04 (0.95 ,1.15)	0.52	1.01 (0.96 ,1.05)	0.00	0.98 (0.92 ,1.05)	0.00
**Post-menopausal Breast**	1.00 (0.97 ,1.04)	0.00	1.04 (1.00 ,1.09)	0.29	1.07 (1.02 ,1.12)	0.46	1.08 (1.03 ,1.14)	0.52	1.10 (1.03 ,1.18)	0.74	1.12 (1.02 ,1.22)	0.79	1.11 (1.09 ,1.14)	0.00	1.12 (1.09 ,1.16)	0.00

* Multivariable adjustment for baseline age, race, alcohol, smoking and hormone replacement therapy (in women).
Green – significant positive interaction. Orange – significant inverse interaction.
Abbreviations: OBR, obesity-related; NOBR, non-obesity related; CI, confidence interval; HR, hazard ratio; BMI, body mass index; MV, multivariable.

Table S14: Hazard ratio of interactions by age on the BMI-cancer link at ages 30 to 65 in (A) men and (B) women in the subgroup with at least 1 BMI measurement, ABACus 2.

(A)

	MV-adjusted HR (95% CI) per 5 unit BMI (kg/m^2)															
	Men															
	Age 30	I^2	Age 35	I^2	Age 40	I^2	Age 45	I^2	Age 50	I^2	Age 55	I^2	Age 60	I^2	Age 65	I^2
All Cancers	1.00 (0.98 ,1.02)	0.00	1.01 (0.99 ,1.03)	0.00	1.01 (0.99 ,1.03)	0.00	1.00 (0.99 ,1.02)	0.00	1.00 (0.99 ,1.02)	0.00	0.99 (0.97 ,1.00)	0.00	0.99 (0.95 ,1.03)	0.73	1.01 (0.94 ,1.07)	0.88
OBR-cancers	1.05 (1.01 ,1.09)	0.00	1.07 (1.02 ,1.13)	0.00	1.07 (1.01 ,1.13)	0.29	1.05 (1.00 ,1.11)	0.36	1.03 (0.99 ,1.07)	0.00	1.00 (0.96 ,1.03)	0.00	0.95 (0.92 ,0.99)	0.00	0.95 (0.88 ,1.02)	0.40
NOBR-cancers	0.99 (0.97 ,1.02)	0.00	1.00 (0.98 ,1.02)	0.00	1.00 (0.98 ,1.02)	0.00	1.00 (0.97, 1.04)	0.38	1.01 (0.97 ,1.04)	0.46	1.01 (0.96 ,1.07)	0.71	1.01 (0.96 ,1.07)	0.79	1.04 (0.94 ,1.15)	0.92
NOBR cancers excluding lung and prostate	1.00 (0.97 ,1.03)	0.00	1.01 (0.98 ,1.04)	0.00	1.01 (0.98 ,1.04)	0.00	0.98 (0.95 ,1.01)	0.08	1.00 (0.92 ,1.09)	0.55	0.96 (0.93 ,0.98)	0.33	0.95 (0.89 ,1.01)	0.45	0.95 (0.87 ,1.04)	0.57
Specific cancer sites																
Colorectal	1.04 (0.98 ,1.11)	0.00	1.09 (1.02 ,1.15)	0.00	1.09 (1.03 ,1.15)	0.16	1.07 (1.01 ,1.13)	0.00	1.05 (0.99 ,1.11)	0.00	1.01 (0.95 ,1.06)	0.00	0.97 (0.92 ,1.02)	0.00	0.97 (0.90 ,1.04)	0.00
Pancreas	1.07 (0.96 ,1.19)	0.00	1.11 (1.00 ,1.23)	0.00	1.11 (1.00 ,1.22)	0.00	1.07 (0.97 ,1.18)	0.00	1.04 (0.95 ,1.14)	0.00	1.00 (0.91 ,1.10)	0.00	0.96 (0.87 ,1.05)	0.00	0.92 (0.80 ,1.06)	0.00
Kidney	1.02 (0.92 ,1.14)	0.00	1.04 (0.93 ,1.16)	0.00	1.04 (0.94 ,1.15)	0.00	1.02 (0.92 ,1.13)	0.00	1.00 (0.91 ,1.10)	0.00	0.97 (0.88 ,1.07)	0.00	0.94 (0.85 ,1.03)	0.10	0.91 (0.82 ,1.02)	0.00
Lung	0.96 (0.90 ,1.01)	0.00	0.92 (0.88 ,0.98)	0.00	0.89 (0.85 ,0.94)	0.00	0.87 (0.83 ,0.92)	0.16	0.87 (0.83 ,0.91)	0.42	0.87 (0.83 ,0.91)	0.34	0.87 (0.80 ,0.95)	0.39	0.91 (0.79 ,1.03)	0.61
Prostate	1.00 (0.97 ,1.03)	0.00	1.01 (0.98 ,1.04)	0.00	1.02 (0.99 ,1.05)	0.00	1.04 (0.99 ,1.08)	0.2	1.04 (1.01 ,1.08)	0.05	1.06 (1.02 ,1.1)	0.21	1.08 (1.01 ,1.14)	0.62	1.12 (1.01 ,1.23)	0.81

* Multivariable adjustment for baseline age, race, alcohol, smoking and hormone replacement therapy (in women).
Green – significant positive interaction. Orange – significant inverse interaction.
Abbreviations: OBR, obesity-related; NOBR , non-obesity related; CI, confidence interval; HR, hazard ratio; BMI, body mass index; MV, multivariable.

(B)

Outcomes	MV-adjusted HR (95% CI) per 5 unit BMI (kg/m²)															
	Women															
	Age 30	I^2	Age 35	I^2	Age 40	I^2	Age 45	I^2	Age 50	I^2	Age 55	I^2	Age 60	I^2	Age 65	I^2
**All Cancers**	1.01 (0.99,1.03)	0.00	1.02 (1.00,1.04)	0.29	1.02 (0.98,1.06)	0.70	1.03 (0.97,1.09)	0.84	1.03 (0.97,1.09)	0.85	1.02 (0.96,1.09)	0.85	1.02 (0.96,1.09)	0.86	1.00 (0.96,1.05)	0.73
**OBR-cancers**	1.03 (1.00,1.05)	0.00	1.05 (1.01,1.08)	0.57	1.05 (1.00,1.10)	0.72	1.05 (0.99,1.11)	0.81	1.04 (0.98,1.11)	0.84	1.04 (0.97,1.11)	0.83	1.03 (0.96,1.12)	0.85	1.02 (0.95,1.10)	0.82
**NOBR-cancers**	0.98 (0.95,1.01)	0.00	0.97 (0.94,1.01)	0.00	0.97 (0.93,1.01)	0.42	0.98 (0.92,1.04)	0.73	0.98 (0.92,1.05)	0.76	0.99 (0.92,1.06)	0.82	1.00 (0.92,1.09)	0.84	0.99 (0.92,1.06)	0.78
**NOBR cancers excluding lung**	0.99 (0.96,1.02)	0.00	0.98 (0.95,1.01)	0.00	0.98 (0.94,1.01)	0.16	1.00 (0.92,1.08)	0.62	1.00 (0.92,1.09)	0.68	1.02 (0.92,1.13)	0.78	1.01 (0.92,1.11)	0.73	0.96 (0.92,1.01)	0.40
**Specific cancer sites**																
**Colorectal**	1.01 (0.95,1.06)	0.00	1.00 (0.95,1.05)	0.00	0.99 (0.94,1.04)	0.00	0.97 (0.93,1.02)	0.00	0.96 (0.92,1.01)	0.30	0.95 (0.9,0.99)	0.00	0.93 (0.88,0.99)	0.51	0.96 (0.81,1.14)	0.75
**Pancreas**	1.00 (0.90,1.10)	0.00	0.99 (0.90,1.08)	0.00	0.97 (0.89,1.07)	0.00	0.95 (0.87,1.04)	0.00	0.94 (0.86,1.03)	0.00	0.93 (0.85,1.01)	0.00	0.92 (0.84,1.00)	0.00	0.9 (0.82,1.00)	0.00
**Kidney**	1.04 (0.94,1.15)	0.00	1.04 (0.95,1.15)	0.00	1.02 (0.93,1.12)	0.00	0.99 (0.90,1.08)	0.09	0.95 (0.87,1.04)	0.09	0.90 (0.83,0.99)	0.00	0.88 (0.80,0.96)	0.05	0.83 (0.74,0.92)	0.00
**Lung**	0.96 (0.91,1.02)	0.00	0.95 (0.88,1.02)	0.33	0.94 (0.87,1.03)	0.60	0.95 (0.86,1.04)	0.71	0.96 (0.87,1.05)	0.76	0.96 (0.88,1.06)	0.75	0.99 (0.89,1.11)	0.80	1.03 (0.91,1.17)	0.81
**Endometrial**	1.06 (1.00,1.12)	0.00	1.07 (0.97,1.18)	0.62	1.09 (0.91,1.31)	0.78	1.07 (0.87,1.33)	0.83	1.03 (0.85,1.24)	0.80	1.00 (0.82,1.22)	0.80	0.97 (0.82,1.15)	0.72	0.88 (0.83,0.94)	0.40
**Ovary**	0.99 (0.91,1.08)	0.00	1.00 (0.92,1.08)	0.00	0.99 (0.91,1.07)	0.00	0.98 (0.91,1.06)	0.00	0.98 (0.91,1.06)	0.00	0.98 (0.91,1.06)	0.00	0.96 (0.89,1.04)	0.00	0.94 (0.86,1.02)	0.00
**Post-menopausal Breast**	1.04 (1.01,1.08)	0.00	1.09 (1.05,1.13)	0.00	1.12 (1.08,1.16)	0.00	1.13 (1.10,1.17)	0.00	1.15 (1.11,1.18)	0.00	1.16 (1.12,1.20)	0.00	1.17 (1.13,1.21)	0.00	1.18 (1.13,1.22)	0.00

* Multivariable adjustment for baseline age, race, alcohol, smoking and hormone replacement therapy (in women).
Green – significant positive interaction. Orange – significant inverse interaction.
Abbreviations: OBR, obesity-related; NOBR, non-obesity related; CI, confidence interval; HR, hazard ratio; BMI, body mass index; MV, multivariable.

- Supplementary figures

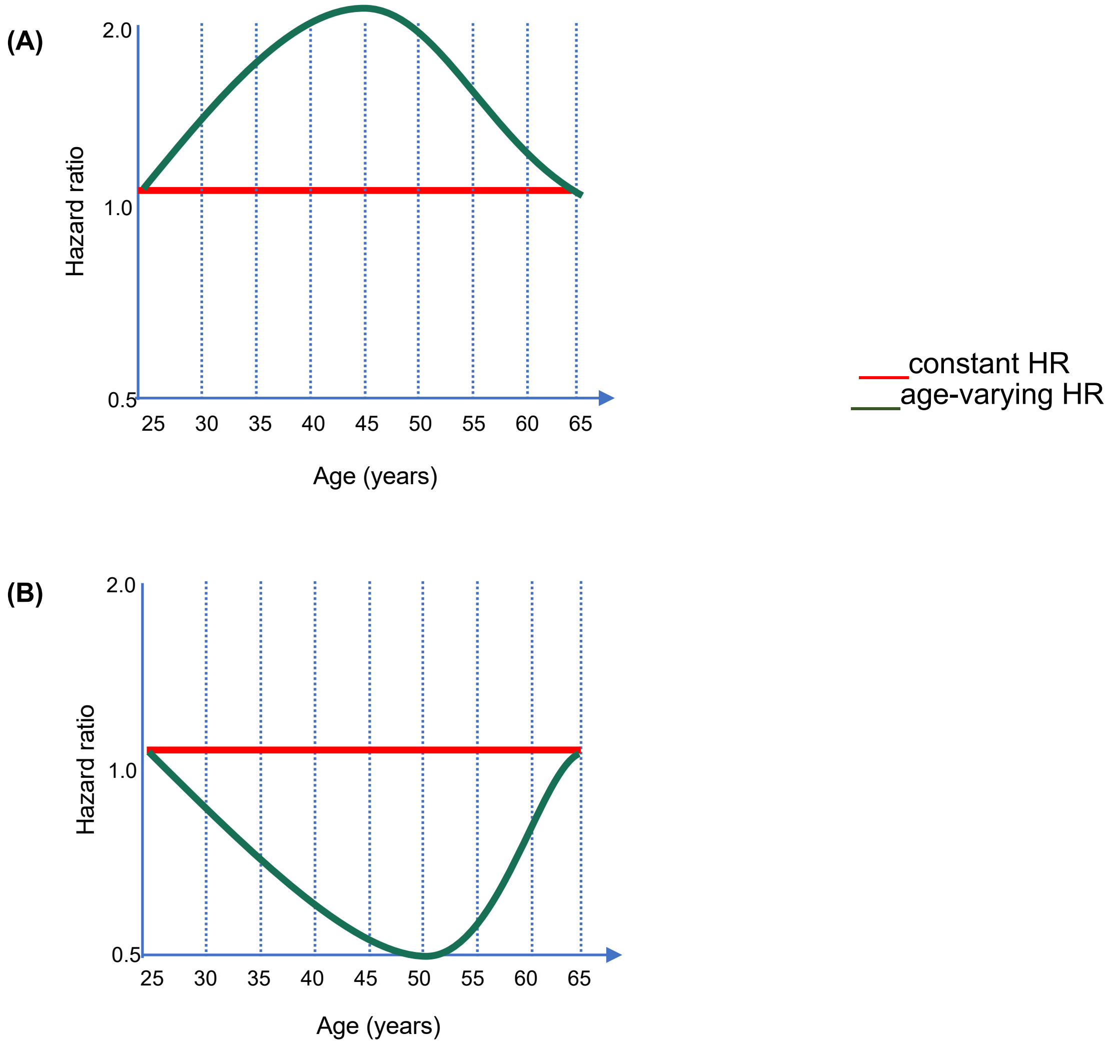

Figure S1: Demonstrating a hypothetical sensitive period analysis of the hazard ratio between per 5-unit BMI exposure and the related cancer risk across adulthood.

The red line is the age-constant hazard ratio, and the green line is the age-varying hazard, which includes an interaction between BMI and age. The x-axis refers to the age at BMI assessment. The y-axis is on a log scale. (A) shows a hypothetical sensitive age period of the BMI-cancer link at age 45, where excess BMI exposure was associated with significantly greater cancer risk compared with other ages, and (B) shows a hypothetical sensitive age period of the BMI-cancer link at age 50 with lower cancer risk per 5-unit excess BMI exposure compared with other ages.

Abbreviations: AOI, age of interest; BMI, body mass index; HR, hazard ratio

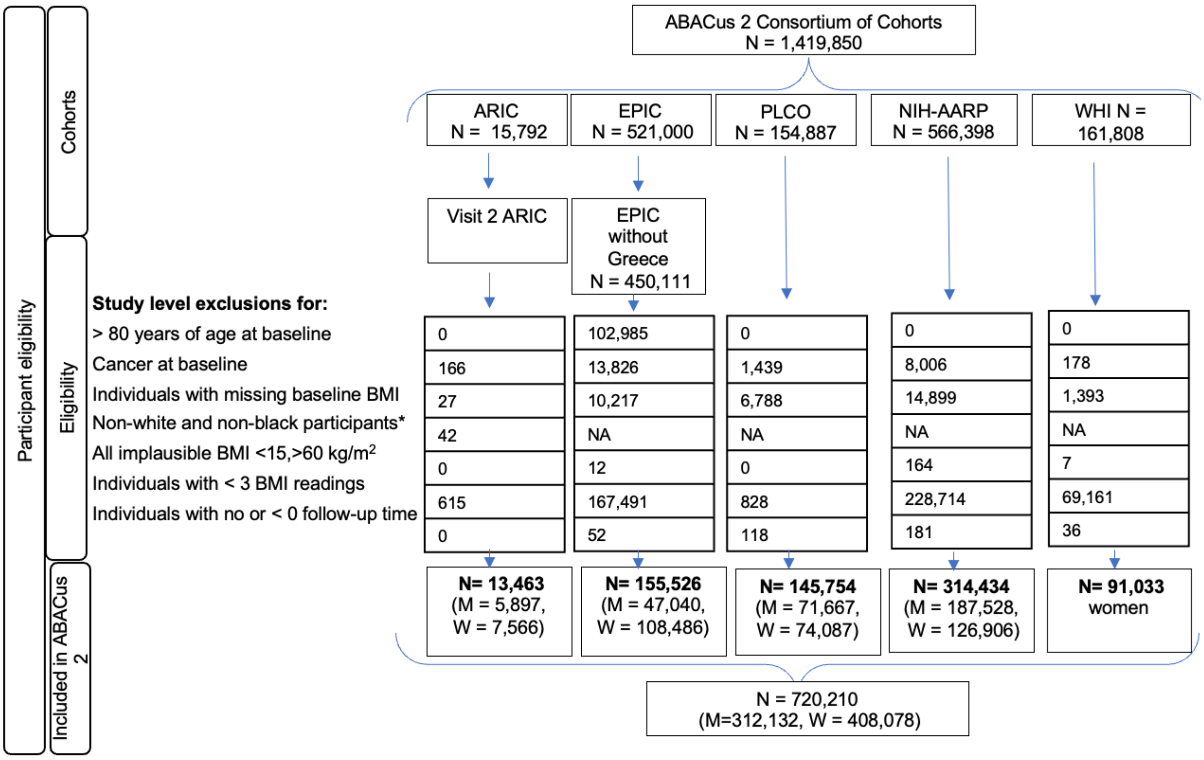

Figure S2: **ABACus 2 Consortium Participant flow diagram. BMI-related exclusion criteria were observational level exclusions but resulted in individual exclusions if none of the BMI readings were within the clinically plausible range.** Figure reproduced from (16).

Abbreviations: ARIC, Atherosclerosis Risk in Communities (Study); EPIC, European Prospective Investigation into Cancer and Nutrition (Study); PLCO, Prostate, Lung, Colorectal and Ovarian Cancer (Screening Trial); NIH-AARP, NIH-AARP Diet and Health Study; WHI, Women's Health Initiative; BMI, body mass index; N, number of participants; M, men; W, women; HRT, hormone replacement therapy.

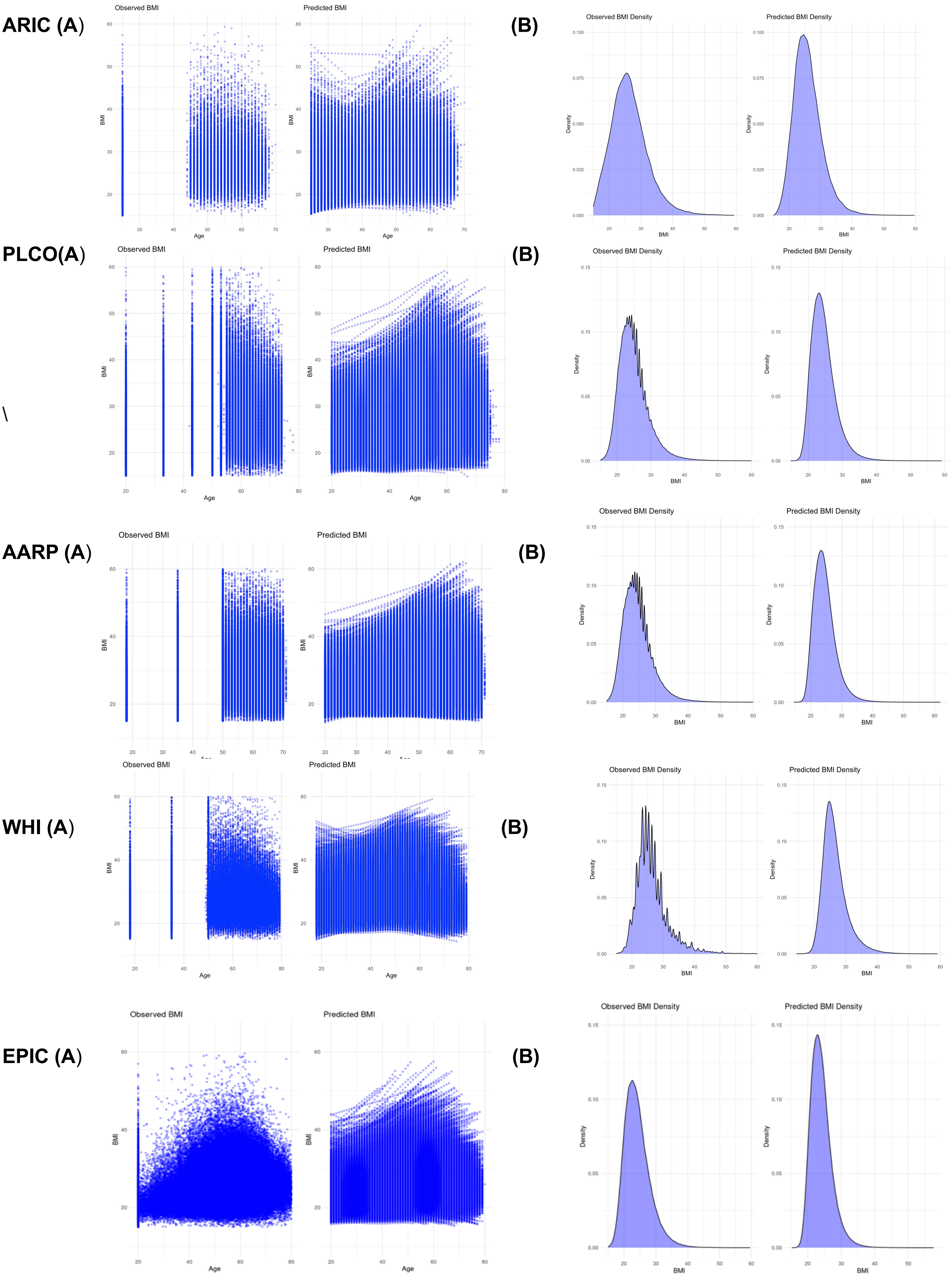

Figure S3: (A) distribution and (B) density of observed and predicted BMI for each cohort.

(A)

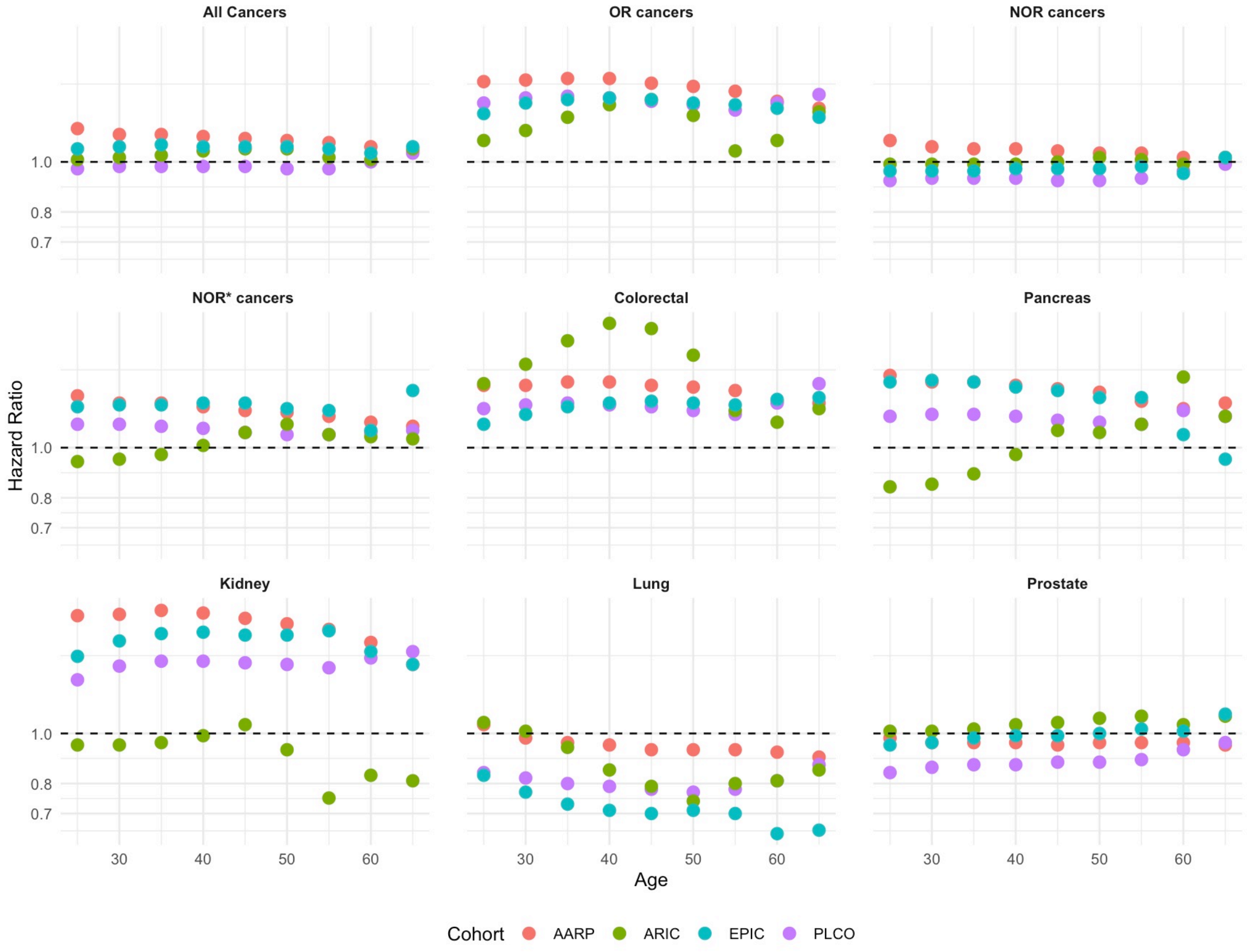

All Cancers
OR cancers
NOR cancers
NOR* cancers
Colorectal
Pancreas
Kidney
Lung
Prostate
Hazard Ratio
1.0
0.8
0.7
30
40
50
60
Age
Cohort
AARP
ARIC
EPIC
PLCO

(B)

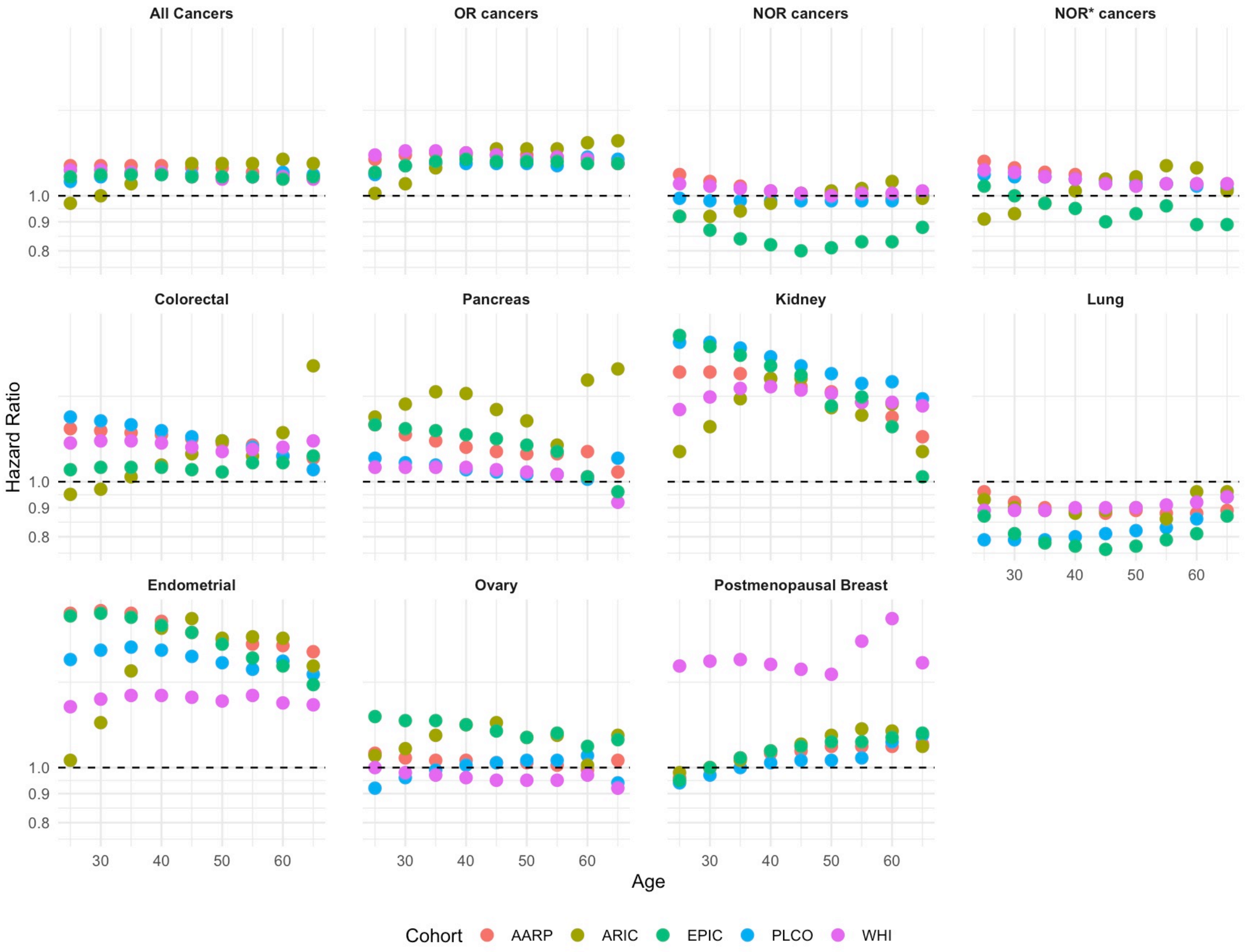

Figure S4: Hazard ratios for cancer incidence per 5-unit BMI exposure at ages 30 to 65 in (A) men and (B) women in the subgroup with at least 3 BMI measurements, ABACus 2.
*Multivariable adjustment for baseline age, race, alcohol, smoking and hormone replacement therapy (in women).
NB: Immortal time bias has been accounted for across all age periods of interest.
Abbreviations: OR, obesity-related; NOR, non-obesity related; CI, confidence interval; HR, hazard ratio; BMI, body mass index; MV, multivariable; EPIC, European Prospective Investigation into Cancer and Nutrition; NIH-AARP, Diet and Health Study; PLCO, Prostate, Lung, Colorectal, Ovarian Cancer Screening Trial; WHI, Women's Health Initiative; ARIC, Atherosclerosis Risk in Communities study

__**Sensitivity Analysis with BMI predicted in the subgroup with at least 1 BMI measurement**__

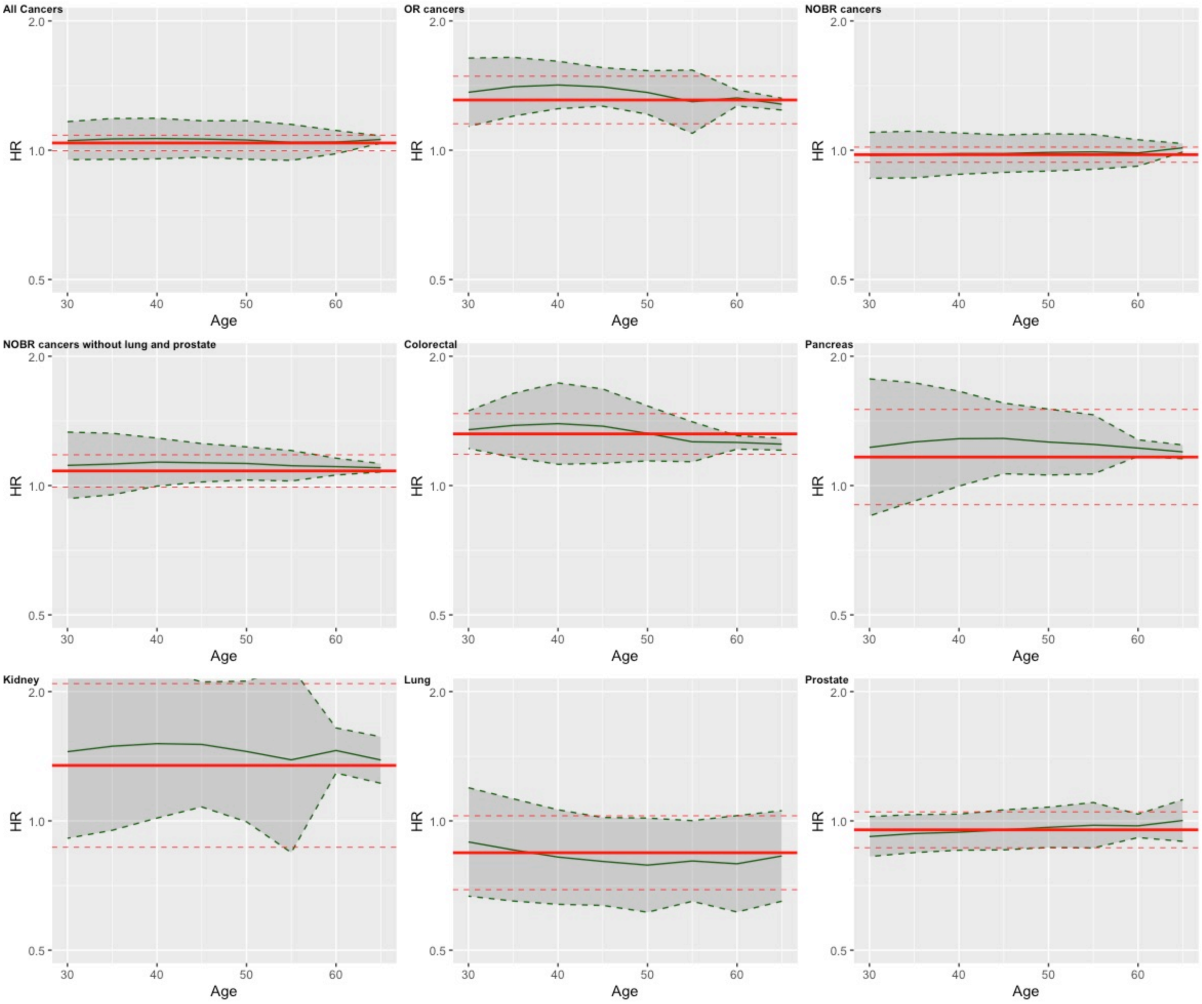

Figure S5: Hazard ratios of cancers per 5-unit BMI (kg/m^2) exposure at ages 30 to 65 for men for combined cancer subgroups and by cancer type over adulthood, ABACus 2. The red line is the age-constant hazard ratio for cancer incidence per 5 unit BMI exposure at ages 30 to 65 (95% CI: dashed red lines), and the green line is the age-varying hazard ratio (which includes an interaction between BMI and age) for cancer incidence per 5 unit BMI exposure at ages 30 to 65 (95% CI: grey ribbon). The x-axis refers to the age at BMI assessment. The y-axis is on a log scale. Multivariable adjustment for baseline age, race, alcohol and smoking.

Abbreviations: OBR, obesity-related cancers; NOBR, non-obesity-related cancers, CI, confidence interval.

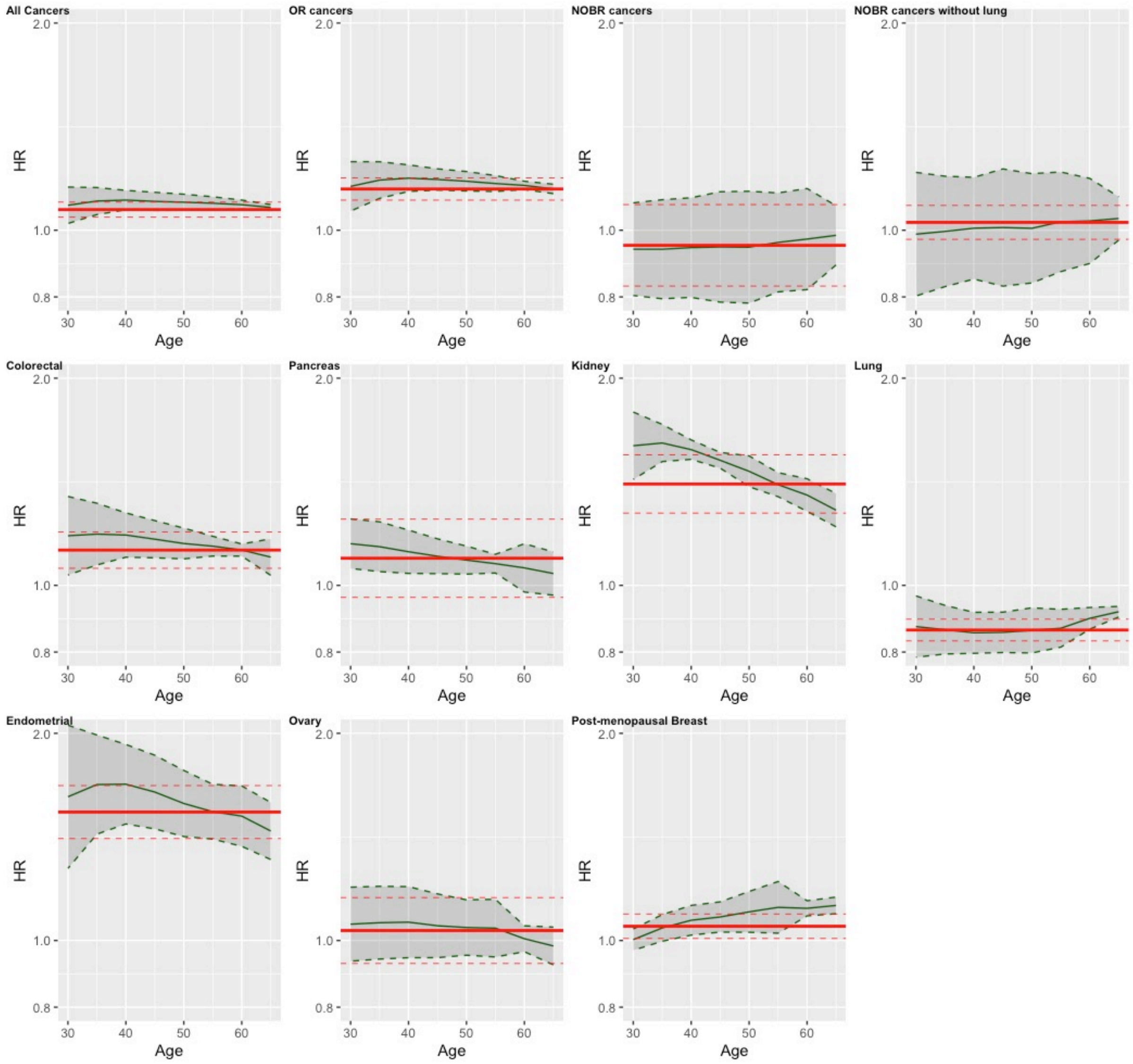

Figure S6: Hazard ratios for cancer incidence per 5-unit BMI (kg/m^2) exposure at ages 30 to 65 for women, ABACus 2. The red line is the age-constant hazard ratio for cancer incidence per 5 unit BMI exposure at ages 30 to 65 (95% CI: dashed red lines), and the green line is the age-varying hazard ratio (which includes an interaction between BMI and age) for cancer incidence per 5 unit BMI exposure at ages 30 to 65 (95% CI: grey ribbon). The x-axis refers to the age at BMI assessment. The y-axis is on a log scale. Multivariable adjustment for baseline age, race, alcohol, smoking and hormone replacement therapy (in women). **Abbreviations**: OBR, obesity-related cancers; NOBR, non-obesity-related cancers, CI, confidence interval.

Supplementary references

1. Eliassen AH, Colditz GA, Rosner B, Willett WC, Hankinson SE. Adult weight change and risk of postmenopausal breast cancer. J Am Med Assoc. 2006 Jul 12;296(2):193–201.
2. Leitzmann MF, Koebnick C, Danforth KN, Brinton LA, Moore SC, Hollenbeck AR, et al. Body mass index and risk of ovarian cancer. Cancer. 2009 Feb 2;115(4):812–22.
3. Beebe-Dimmer JL, Colt JS, Ruterbusch JJ, Keele GR, Purdue MP, Wacholder S, et al. Body mass index and renal cell cancer: The influence of race and sex. Epidemiology. 2012 Nov;23(6):821–8.
4. Schoemaker MJ, Nichols HB, Wright LB, Brook MN, Jones ME, O'Brien KM, et al. Association of Body Mass Index and Age with Subsequent Breast Cancer Risk in Premenopausal Women. JAMA Oncol. 2018 Nov 1;4(11).
5. Hopper JL, Dite GS, MacInnis RJ, Liao Y, Zeinomar N, Knight JA, et al. Age-specific breast cancer risk by body mass index and familial risk: Prospective family study cohort (ProF-SC). Breast Cancer Res. 2018 Nov 3;20(1):1–11.
6. Renehan AG, Pegington M, Harvie MN, Sperrin M, Astley SM, Brentnall AR, et al. Young adulthood body mass index, adult weight gain and breast cancer risk: the PROCAS Study (United Kingdom). Br J Cancer. 2020 May 12;122(10):1552–61.
7. Furer A, Afek A, Sommer A, Keinan-Boker L, Derazne E, Levi Z, et al. Adolescent obesity and midlife cancer risk: a population-based cohort study of 2·3 million adolescents in Israel. Lancet Diabetes Endocrinol. 2020 Mar 1;8(3):216–25.
8. Genkinger JM, Wu K, Wang M, Albanes D, Black A, van den Brandt PA, et al. Measures of body fatness and height in early and mid-to-late adulthood and prostate cancer: risk and mortality in The Pooling Project of Prospective Studies of Diet and Cancer. Ann Oncol. 2020 Jan 1;31(1):103–14.
9. Li H, Boakye D, Chen X, Jansen L, Chang-Claude J, Hoffmeister M, et al. Associations of Body Mass Index at Different Ages With Early-Onset Colorectal Cancer. Gastroenterology. 2022 Apr 1;162(4):1088–1097.e3.
10. Loomans-Kropp HA, Umar A. Analysis of Body Mass Index in Early and Middle Adulthood and Estimated Risk of Gastrointestinal Cancer. JAMA Netw open. 2023 May 10;6(5):e2310002.
11. The ARIC investigators. The atherosclerosis risk in community (ARIC) study: Design and objectives. Am J Epidemiol. 1989 Apr 1;129(4):687–702.
12. Haftenberger M, Lahmann P, Panico S, Gonzalez C, Seidell J, Boeing H, et al. Overweight,obesity and fat distribution in 50- to 64-year-old participants in the European Prospective Investigationinto Cancer and Nutrition (EPIC). Public Health Nutr. 2002;5(6b):1147–62.
13. Arnold M, Jiang L, Stefanick ML, Johnson KC, Lane DS, LeBlanc ES, et al. Duration ofAdulthood Overweight, Obesity, and Cancer Risk in the Women's Health Initiative: A LongitudinalStudy from the United States. Prentice A, editor. PLoS Med. 2016 Aug 16;13(8):e1002081.
14. Kelly SP, Graubard BI, Andreotti G, Younes N, Cleary SD, Cook MB. Prediagnostic bodymass index trajectories in relation to prostate cancer incidence and mortality in the PLCO cancerscreening trial. J Natl Cancer Inst. 2017 Mar 1;109(3).
15. Klein JP, Moeschberger ML. Survival Analysis. 2003; 16.Hawwash NK, Sperrin M, Martin GP,Sinha R, Matthews CE, Ricceri F, et al. Excess weight by degree and duration and cancer risk (ABACus2 consortium): a cohort study and individual participant data meta-analysis.eClinicalMedicine. 2024 Dec 1;78.
16. Hawwash NK, Sperrin M, Martin GP, Sinha R, Matthews CE, Ricceri F, et al. Excess weight bydegree and duration and cancer risk (ABACus2 consortium): a cohort study and individual participantdata meta-analysis. eClinicalMedicine. 2024 Dec 1;78.
